# Characterization of zygotic genome activation-dependent maternal mRNA clearance in mouse

**DOI:** 10.1093/nar/gkz1111

**Published:** 2019-11-28

**Authors:** Qian-Qian Sha, Ye-Zhang Zhu, Sen Li, Yu Jiang, Lu Chen, Xiao-Hong Sun, Li Shen, Xiang-Hong Ou, Heng-Yu Fan

**Affiliations:** 1 MOE Key Laboratory for Biosystems Homeostasis & Protection and Innovation Center for Cell Signaling Network, Life Sciences Institute, Zhejiang University, Hangzhou 310058, China; 2 Fertility Preservation Laboratory, Reproductive Medicine Center, Guangdong Second Provincial General Hospital, Guangzhou 510317, China; 3 Key Laboratory of Reproductive Dysfunction Management of Zhejiang Province; Assisted Reproduction Unit, Department of Obstetrics and Gynecology, Sir Run Run Shaw Hospital, School of Medicine, Zhejiang University, Hangzhou 310016, China

## Abstract

An important event of the maternal-to-zygotic transition (MZT) in animal embryos is the elimination of a subset of the maternal transcripts that accumulated during oogenesis. In both invertebrates and vertebrates, a maternally encoded mRNA decay pathway (M-decay) acts before zygotic genome activation (ZGA) while a second pathway, which requires zygotic transcription, subsequently clears additional mRNAs (Z-decay). To date the mechanisms that activate the Z-decay pathway in mammalian early embryos have not been investigated. Here, we identify murine maternal transcripts that are degraded after ZGA and show that inhibition of *de novo* transcription stabilizes these mRNAs in mouse embryos. We show that YAP1-TEAD4 transcription factor-mediated transcription is essential for Z-decay in mouse embryos and that TEAD4-triggered zygotic expression of terminal uridylyltransferases TUT4 and TUT7 and mRNA 3′-oligouridylation direct Z-decay. Components of the M-decay pathway, including BTG4 and the CCR4-NOT deadenylase, continue to function in Z-decay but require reinforcement from the zygotic factors for timely removal of maternal mRNAs. A long 3′-UTR and active translation confer resistance of Z-decay transcripts to M-decay during oocyte meiotic maturation. The Z-decay pathway is required for mouse embryo development beyond the four-cell stage and contributes to the developmental competence of preimplantation embryos.

## INTRODUCTION

The earliest stages of metazoan embryonic development are controlled by maternal gene products. During the maternal-to-zygotic transition (MZT), developmental control passes from the maternal to the zygotic genome via a combination of two processes: first, the majority of maternal mRNAs is eliminated; second, the zygotic genome becomes transcriptionally active. There is a complex interplay of maternal and zygotic products in regulating both aspects of MZT, thus ensuring timely transfer of developmental control (1–3).

During the MZT in the fruit fly, zebrafish and frog, clearance of these maternal mRNAs is accomplished through the combined action of two degradation activities, one ‘maternal’ and the other ‘zygotic’ (4–6). The former is exclusively composed of maternally encoded products whereas the latter requires zygotic genome activation to produce and/or activate the decay machinery. A subset of RNA-binding proteins accumulated during oogenesis as specific factors to direct the maternal degradation machinery to its target mRNAs (7–9). On the other hand, small RNAs, most notably microRNAs, have been identified as mediators of the zygotically encoded mRNA degradation activity in *Drosophila*, zebrafish, and *Xenopus* (6,10–13). In these model organisms, high-level zygotic genome activation (ZGA) coincides with lengthening and desynchronization of mitoses at the onset of gastrulation, an event known as the ‘mid-blastula transition (MBT)’ (2). However, in mammalian embryos, ZGA occurs as early as the 1–4 cell stage, resulting in a unique ‘pre-blastula transition’ (1,14,15). For example, in the mouse embryo, zygotic transcription is first detected at the late 1-cell stage, whereas the majority of maternal mRNAs are removed by the two-cell stage (16). Gene expression profiling experiments have provided evidence for what are probably the maternal and zygotic degradation activities: a subset of maternal transcripts is quickly degraded following oocyte meiotic resumption, whereas others show later decreases that coincide with ZGA at the two-cell stage.

Recent studies have indicated that the oocyte-expressed MZT licensing factor, BTG4, mediates maternal mRNA degradation in mouse oocytes and zygotes by recruiting the CCR4-NOT deadenylase complex to actively translating transcripts (17–19). CNOT6L, a CCR4-NOT catalytic subunit, is preferentially expressed in mouse oocytes, and mediates meiosis-coupled maternal mRNA decay (20,21). Genomic *Btg4* or *Cnot6l* knockout mice are healthy, but the females are infertile because zygotes derived from their oocytes have severe MZT defects (17,20). In addition, oocyte-derived terminal uridylyltransferases TUT4 and TUT7 (TUT4/7) are crucial for mRNA clearance during mouse oogenesis (22). The RNA m^6^A reader YTHDF2 is required during oocyte maturation for post-transcriptional regulation of transcript dosage for early zygotic development (23). Collectively, these findings reveal the existence, components and functional importance of the maternal factor-mediated mRNA decay (M-decay) pathway in the mammalian MZT. However, whether the zygotic decay (Z-decay) pathway also has a key function in mammalian embryo development has not been investigated.

In this study, we defined and characterized ZGA-dependent maternal mRNA clearance during the mouse MZT and demonstrated that the 3′-UTR length and translational activity of a given maternal transcript determines whether it undergoes M-decay or Z-decay. YAP1- and TEAD4-mediated zygotic transcription is crucial for activation of the Z-decay pathway in mouse embryos. In particular, TEAD4-triggered zygotic *Tut4/7* expression and mRNA 3′-oligouridylation play a key role in Z-decay, and collaborate with the maternal mRNA deadenylation machinery including BTG4 and CCR4-NOT. Activity of this Z-decay pathway is required for mouse embryo development beyond the four-cell stage and contributes to the developmental potential of preimplantation embryos.

## MATERIALS AND METHODS

### Animals

All the used mouse strains were from a C57B6 background. Wild type C57BL6 mice were obtained from the Zhejiang Academy of Medical Science, China. The experimental protocols involving mice were approved by the Zhejiang University Institutional Animal Care and Research Committee (Approval # ZJU20170014), and mouse care and use was performed in accordance with the relevant guidelines and regulations.

### Oocyte culture

The 21–23-day-old female mice were injected with 5 IU of PMSG and were humanely euthanized after 44 h. Oocytes at the GV stage were harvested in M2 medium (M7167; Sigma-Aldrich) and cultured in mini-drops of M16 medium (M7292; Sigma-Aldrich) covered with mineral oil (M5310; Sigma-Aldrich) at 37°C in a 5% CO_2_ atmosphere.

### Superovulation and fertilization

Female mice (21–23-day-old) were intraperitoneally injected with 5 IU of PMSG (Ningbo Sansheng Pharmaceutical Co., Ltd, P.R. China). After 44 h, mice were injected with 5 IU of hCG (Ningbo Sansheng Pharmaceutical Co., Ltd, P.R. China). After an additional 16 h, mature oocytes were harvested from the oviducts. To obtain early embryos, female mice were mated with 10–12-week-old WT males. Successful mating was confirmed by the presence of vaginal plugs. Embryos were harvested from oviducts at the indicated time points after hCG injection.

### Treatment of mouse embryos with α-amanitin

Zygotes were collected from oviducts after hCG 28 h. To inhibit transcription in early embryos, zygotes were cultured in KSOM medium supplemented with α-amanitin (25 ng/μl, Sigma-Aldrich) for about 16 h. After the culture, morphologically normal two-cell embryos were collected for additional experiments.

### EU incorporation assay

Embryos were cultured in KSOM medium with 100 μM 5-ethynyl uridine (EU) for 2 h. Fixation, permeabilization, and staining were performed using the Click-iT^®^ RNA Alexa Fluor^®^ 488 Imaging Kit (Thermo, 48 C10329) according to the manufacturer's protocol. Imaging of embryos was performed on a Zeiss LSM710 confocal microscope.

### Microinjection of zygotes

All injections were performed using an Eppendorf transferman NK2 micromanipulator. Denuded zygotes were incubated in M2 medium and microinjected with 5–10 pl samples per zygote. The concentration of all microinjected RNAs was adjusted to 500 ng/μl. After microinjection, zygotes were washed and cultured in KSOM medium at 37°C with 5% CO_2_.

### *In vitro* transcription and preparation of mRNAs for microinjection

To prepare mRNAs for microinjection, expression vectors were linearized, and subjected to phenol/chloroform extraction and ethanol precipitation. Linearized DNAs were *in vitro* transcribed using the SP6 message mMACHINE Kit (Invitrogen, AM1340). Transcribed mRNAs were added with poly (A) tails (∼200–250 bp) using the mMACHINE Kit (Invitrogen, AM1350), recovered by lithium chloride precipitation, and resuspended in nuclease-free water.

### *Tut4/7* mRNA depletion by siRNAs

All small RNA(siRNAs) were purchased from RIBOBIO. We targeted each gene with a siRNA pool (two siRNAs per gene), which on average leads to fewer off-target effects and a higher penetrance of phenotypes compared to individual siRNA. All small RNAs were modified with cholesterol to increase their stability. siRNAs targeting different genes were mixed and microinjected at a final concentration of 20 μM with 5 to 10 pl samples per oocyte. Previously published *Tut4* and *Tut7* siRNA sequences were used to knockdown *Tut4/7*. We microinjected the Random sequence (control siRNA1: UGGUUUACAUGUCGACUAATT; control siRNA2: UGGUUUACAUGUUGUGUGATT) cited from a published paper (Chang *et al.*, *Nature*, 2014) as a control to rule out the non-specific effects.

### Poly(A) tail assay

Total RNA was isolated from 100 oocytes or embryos using the RNeasy Mini kit (Qiagen, 74106). P1 (5′-GCGAGCTCCGCGGCCGCGT12-3′) was anchored to oligo(dT) by T4 DNA ligase. Reverse transcription was performed using SuperScript IV (Invitrogen) with oligo (dT) anchored P1. The products were used in a PCR with gene-specific primers P2 (Supplementary Table S1) and the dT anchored primer P1 (5′-GCGAGCTCCGCGGCCGCGT12-3′). The PCR conditions were as follows: 30 s at 95°C, 20 s at 58°C, and 40 s at 72°C for 35 cycles. PCR products were analyzed on a 2% agarose gel.

### Oligo(U) tail assay

Total RNA was isolated from 100 oocytes or embryos using the RNeasy Mini kit (Qiagen, 74106). Reverse transcription was performed using the SuperScript IV (Invitrogen) with oligo (dA_12_) that specifically enriched for uridylated RNA species. Relative uridylated mRNA levels were calculated by normalizing to the levels of encoded exogenous *Gfp* cDNA. The relative expression of mRNA was reversed by the random hexamer-primer. Relative mRNA levels were calculated by normalizing to the levels of endogenous *Gapdh* mRNA (internal control). For each experiment, qPCR was performed in triplicate. Primer sequences are listed in Supplemental Table S1.

### Trim-away

For prompt depletion of a target protein, a Trim-away approach was used as reported recently (24,25). Zygotes were co-injected with *in vitro* transcribed *Flag-Trim21* mRNA (1 μg/μl) and anti-BTG4 antibody (0.75 μg/μl) at 20 h after hCG injection. As the control, zygotes were only injected with *Flag-Trim21* mRNA (1 μg/μl). After microinjection, zygotes were washed and cultured in KSOM medium at 37°C with 5% CO_2_.

### RNA isolation and real-time RT-PCR

Total RNA was extracted using an RNeasy Mini kit (Qiagen, 74106) according to the manufacturer's instructions, and was reversely transcribed using the PrimeScript II 1st strand cDNA Synthesis (Takara, 6210A). A random primer (hexadeoxyribonucleotide mixture; pd(N)6;Takara,3801) (50 μM) was used to guide the reverse transcription. Real-time RT-PCR analysis was performed using a Power SYBR Green PCR Master Mix (Applied Biosystems, Life Technologies) and an Applied Biosystems 7500 Real-Time PCR System. Relative mRNA levels were calculated by normalizing to the levels of endogenous *Gapdh* mRNA (internal control) or encoded exogenous *Gfp* cDNA using Microsoft EXCEL^®^. The relative transcript levels of samples were compared to the control, and the fold-changes are demonstrated. For each experiment, qPCR was performed in triplicate. Primer sequences are listed in Supplementary Table S1.

### Western blot analysis

Oocytes were lysed in SDS loading buffer at 95°C for 5 min. SDS-PAGE, membrane transfer, and antibody incubation were performed following standard procedures using a Mini-PROTEAN Tetra Cell System (Bio-Rad, Hercules, CA, USA). The information of antibodies and dilutions used in this study is provided in Supplementary Table S2.

### RNA-Seq library preparation

Embryos were collected from the mice of indicated genotypes (10 embryos per sample). Each sample was lysed with 4.2 μl lysis buffer (including 0.2 μl 1:1000 diluted ERCC spike-in) and was immediately used for cDNA synthesis using the Smart-seq2 method (Picelli *et al.*, 2014). Briefly, cells were lysed in lysis buffer, and the polyadenylated mRNAs were captured usingby the PolyT primers. After 3 min lysis at 72°C, the Smart-seq2 reverse transcription reactions were performed. After the first-strand reaction, the cDNA wasis amplified using a limited number of cycles (∼13 cycles). Sequencing libraries were constructed from 500 pg of amplified cDNA using the TruePrep DNA Library Prep Kit V2 for Illumina (Vazyme, TD503) according to the manufacturer's instructions. Barcoded libraries were pooled and sequenced on the Illumina HiSeq X Ten platform in the 150 bp paired-end mode.

### RNA-Seq data analysis

RNA-Seq was performed with biological replicates for all samples. Raw reads were trimmed to 50 bp and mapped to the mouse genome (mm9) using Tophat v2.1.1 with default parameters. Only uniquely mapped reads were subsequently assembled into transcripts guided by the reference annotation (UCSC gene models) using Cufflinks v2.2.1. The expression level of each gene was quantified with normalized FPKM (fragments per kilobase of exon per million mapped fragments) and was further normalized with the ERCC spike-in. Briefly, sequencing reads were mapped to ERCC reference to obtain the percentage of ERCC reads in total reads. Then gene expression levels were normalized by multiplying the raw FPKM values by a normalization factor (normalization factor = percentage of ERCC in WT 1Cell/percentage of ERCC rate in the sample). Samples prepared in different batches were normalized by the zygote sample in each batch. Genes with FPKM < 1 in all samples were excluded, and for the remaining genes, all FPKM values smaller than 1 were set to 1 in subsequent analyses. Two-tailed Student's t-test was used to generate statistically significant values between WT and *Tut4/7*-depeleted samples. A summary of RNA-seq data generated in this study is shown in Supplementary Table S3. Other published data sets used in this study were listed in Supplementary Table S4.

### Maternal transcript clustering

Maternal mRNAs with reliable sequence annotation and FPKM > 2 at the GV stage were retained for further analysis. Expression level of each gene were plus one then log2 transformed in the following analysis. Cluster I-IV consists the genes which satisfy the following two formulas:

Cluster I: Expression (GV) > Expression (Zygote) + 1; Expression (Zygote) < Expression (two-cell) + 1.

Cluster II: Expression (GV) < Expression (Zygote) + 1; Expression (GV) > Expression (Zygote) – 1; Expression (Zygote) > Expression (two-cell) + 1.

Cluster III: Expression (GV) > Expression (Zygote) + 1; Expression (Zygote) > Expression (two-cell) + 1.

Cluster IV: Expression (GV) < Expression (Zygote) + 1; Expression (GV) > Expression (Zygote) – 1; Expression (Zygote) < Expression (two-cell) + 1; Expression (Zygote) > Expression (two-cell) – 1.

### Polyribosome-bound RNA isolation

Polyribosomes were isolated from oocytes as reported (20). Fully grown GV oocytes are collected from PMSG-primed (44 h) 23-day-old mice. MII oocytes were collected at 16 after cultured. 500 oocytes of every sample were lysed with PLB (30 mM Tris–HCl at pH 7.5, 100 mM NaCl, 10 mM MgCl_2_, 1% Triton, 1 mM DTT, 0.25 mM Na_3_VO_4_, 20 mM beta-glycerophosphate, 40 U/ml RNase inhibitor (Takara), 100 lg/ml cycloheximide, plus protease inhibitor cocktail). Oocyte lysates were loaded on a 10-ml 15–50% sucrose gradient and centrifuged at 200 000 g for 120 min at 4°C. RNAs were precipitated adding with 1/10 volume of NaAc and 3× volume of ethanol at −80°C. Polyribosome-bound RNAs were purified with RNeasy Mini kit (Qiagen, 74106). Library construction, sequencing, and analyses for polyribosome-bound RNAs was similar to the method used for RNA-seq.

### 3′-UTR analysis

The 3′-UTR sequences of mouse (mm9) were extracted from UCSC Table Browser. The conserved sequences UUUUAU/UUUUAAU and AAUAAA/AUUAAA were used to identify CPEs and PASs, respectively. The lengths of 3′-UTRs and numbers of CPEs and PASs in 3′-UTRs were calculated using an in-house Python script.

### Statistical analysis

Results are presented as means ± S.E.M. Most experiments included at least three independent samples and were repeated at least three times. The results for two experimental groups were compared using two-tailed unpaired Student's *t*-tests. Statistically significant values of *P* < 0.05, *P* < 0.01, and *P* < 0.001 by two-tailed Student's *t*-test are indicated by asterisks (*), (**) and (***) respectively. ‘n.s.’ indicates non-significant.

## RESULTS

### Patterns of maternal mRNA degradation in the mouse

**Figure 1.**
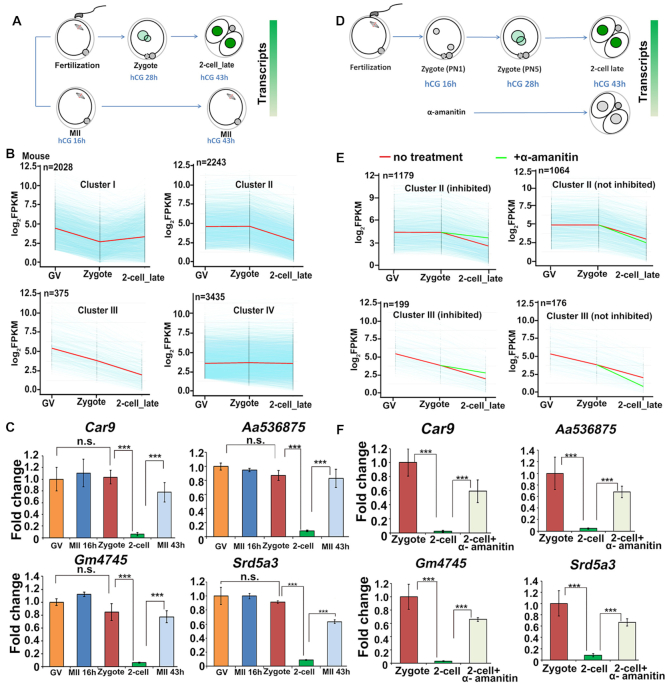
Dynamics of maternal mRNA clearance in mouse preimplantation embryos. (**A**) Illustration of the timepoints when the samples were collected for experiments in (B) and (C). (**B**) Expression pattern of mouse maternal transcripts at GV, zygote, and late two-cell stages. Transcripts with FPKM > 2 in GV oocytes were selected and further analyzed. Each light blue line represents the expression level of one gene, and the middle red line represents the median expression level of the cluster. Zygotes and two-cell embryos were collected from *in vivo* at 28 h and 43 h post-hCG injection. (**C**) RT-qPCR results showing the relative mRNA levels of select transcripts in mouse oocytes and embryos at the timepoints indicated in (A). Error bars, s.e.m. ***P < 0.001 by two-tailed Student's *t*-test. n.s.: non-significant. *n* = 3 biological replicates. (**D**) Illustration showing the treatment of mouse oocytes and embryos for RNA-seq. Zygotes were treated with or without α-amanitin (25 ng/μl), and then cultured to the two-cell stage until 43 h after hCG injection. (**E**) The degradation pattern of maternal transcripts in mouse embryos with or without α-amanitin treatment. Transcripts with FPKM (two-cell)/FPKM (zygote) < 1/2 were selected for analyses. Each light blue line represents the expression level of one gene. The middle red line represents the median expression level of the cluster. The green line represents the median expression level of the cluster after α-amanitin treatment. (**F**) RT-qPCR results showing the relative mRNA levels in mouse zygotes and two-cell embryos, which were treated and collected as illustrated in (D). Error bars, s.e.m. ***P < 0.001 and **P < 0.01 by two-tailed Student's *t*-test. **n** = 3 biological replicates.

To identify patterns of maternal mRNA degradation during the MZT in mouse, we analyzed the maternal transcripts of GV oocytes, zygotes and two-cell embryos (GSE71434 (26)). Maternal mRNAs with reliable sequence annotation and fragments per kilobase of transcript per million mapped reads (FPKM) > 2 at the GV stage were selected and analyzed. Those with significant decreases in mRNA levels of >2-fold between two stages were considered as degraded maternal mRNAs. Among the 8081 maternal transcripts analyzed, 2028, 2243, 375 and 3435 transcripts were respectively categorized into cluster I, cluster II, cluster III and cluster IV. (Figure 1A and B). Cluster I was comprised of mRNAs that were degraded during oocyte maturation (from GV to zygote). In contrast, Cluster II represented the maternal mRNAs that showed no changes in their levels before fertilization but dramatically decreased from the zygote to the two-cell stage. Maternal mRNAs that were either continuously degraded or remained stable from the GV to the two-cell stage were categorized into Clusters III and IV, respectively. Since ZGA occurs at the late zygote to two-cell stage, we suggest that the degradation of maternal mRNAs in Clusters II and III were candidates to be ZGA-dependent.

Usually, 40–44 h are required for the induction of ovulation (hCG injection) to the formation of two-cell embryos, if the ovulated MII oocytes are fertilized *in vivo* (Figure 1A) or *in vitro*. Therefore, the degradation of these maternal transcripts after fertilization could either be time-dependent or fertilization-dependent, or both. The quantitative RT-PCR (RT-qPCR) results in Figure 1C demonstrate that some murine maternal transcripts are stable during oocyte maturation and fertilization but are rapidly degraded at the two-cell stage. We compared the levels of these maternal transcripts in two-cell embryos with those in oocytes arrested at a prolonged MII stage without fertilization. Both the two-cell embryos and MII oocytes were collected at 43 h after hCG injection (illustrated in Figure 1A). The levels of maternal mRNAs in these aged MII oocytes were lower than those in MII oocytes and zygotes harvested at an earlier time point (16 h post-hCG) but were notably higher than those in time-matched two-cell embryos (43 h post-hCG). This phenomenon suggested that the clearance of some maternal transcripts is partially ZGA-dependent.

To assess whether all Cluster IV transcripts remained stable beyond the two-cell stage or whether a subset were degraded after this timepoint, we also analyzed their levels at the four-cell stage (Supplementary Figure S1A). This analysis indicated that over a third of the Cluster IV transcripts were, in fact, degraded between the two-cell and four-cell stages (Supplementary Figure S1A). Thus, while previous studies indicated that maternal mRNAs are mostly degraded by the end of the two-cell stage in mouse (1), our new analyses show that a significant number of maternal mRNAs were kept relatively stable until the late two-cell stage, but were degraded at the four-cell stage. This category of maternal mRNAs includes those encoding the MZT licensing factor BTG4 and RNA deadenylases such as CNOT7 and PAN2 (Supplementary Figure S1B).

Although we observed that maternal mRNA in cluster IV is not degraded from the GV to two-cell stage, it is possible that some of these maternal transcripts have been degraded but they look like stable because they have been synthesized from the embryonic genome: the transcripts from the embryonic genome compensate for the loss of the maternal transcripts.

### Identification of ZGA-dependent maternal mRNA degradation during the mouse MZT

As a subpopulation of mouse maternal mRNAs in Cluster II and Cluster III was degraded at the late two-cell stage, which coincides with ZGA in mouse oocytes, we further investigated whether degradation of these maternal mRNAs was ZGA-dependent. Zygotes were treated with the RNA polymerase II inhibitor, α-amanitin, to inhibit transcription before ZGA (Figure 1D). α-Amanitin-treated zygotes were able to develop to two-cell embryos but not further (14). To confirm that drug treatment was effective, we labelled newly synthesized RNAs with 5-ethynyl uridine (EU), which was added to the culture medium 2 h before fixation of the two-cell embryos. Strong EU signals were detected in the nuclei of control embryos but not in embryos treated with α-amanitin (Supplementary Figure S2A). We also analyzed two zygotic mRNAs that were identified by RNA sequencing analysis to be transcribed at the two-cell stage (*Gucala* and *Npl*) and two key ZGA factors (*Dux* and *Zscan4c*). RT-qPCR demonstrated that α-amanitin blocked the transcriptional activation of these genes in two-cell embryos (Supplementary Figure S2B and C).

Transcriptome analysis of mouse maternal mRNAs showed that the median levels of Cluster II and III mRNAs were decreased by >2-fold from the zygote to the two-cell stages. However, α-amanitin treatment blocked the degradation of half of the transcripts in Cluster II (Figure 1E) while, in Cluster III, 199 of 375 transcripts were stabilized during development from the zygote to two-cell stage in the absence of ZGA (Figure 1E). By RT-qPCR, we demonstrated that representative maternal transcripts were degraded during development from the zygote to two-cell stage, and α-amanitin partially inhibited the degradation of these transcripts (Figure 1F). This observation was consistent with the RNA-seq results and confirmed that degradation of a subpopulation of maternal mRNAs is ZGA-dependent in mouse.

### Maternal YAP1 and zygotic TEAD4 are involved in the Z-decay pathway of mouse embryos

**Figure 2.**
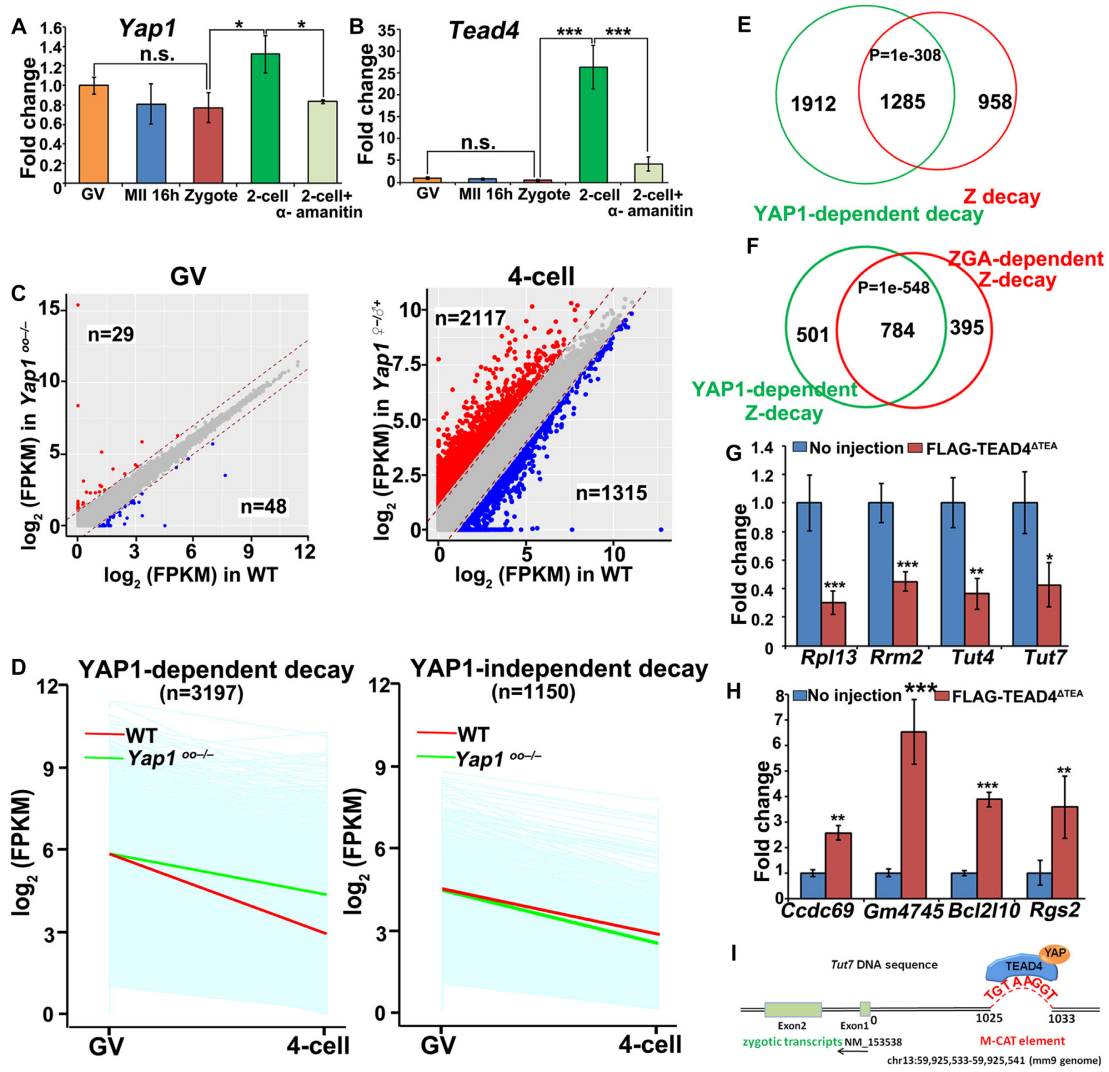
Role of maternal YAP1 and zygotic TEAD4 in the Z-decay pathway of mouse embryos. (**A**, **B**): RT-qPCR results showing the relative mRNA levels of *Yap1* (A) and *Tead4* (B) in GV oocytes, MII oocytes after 16 h of hCG injection, zygotes, two-cell embryos, and α-amanitin-treated embryos. The embryos were treated with α-amanitin (25 ng/μl), and then cultured to the 2-cell stage until 43 h after the hCG injection. Error bars, s.e.m. *P < 0.05 and ***P < 0.001 by two-tailed Student's *t*-test. n.s.: non-significant. *n* = 3 biological replicates. (**C**) Scatter plot comparing transcripts between the WT and *Yap1^–/–^* oocytes (at the GV stage) and the four-cell embryos derived from these oocytes. Transcripts decreased or increased by >2-fold in *Yap1^–/–^* samples were highlighted in blue or red, respectively. (**D**) Degradation pattern of maternal transcripts in mouse embryos with or without maternal *Yap1* knockout. Transcripts with FPKM (GV) > 2; FPKM (four-cell/GV) < 1/2 were selected for the analyses. Each light blue line represents the expression level of one gene. The middle red line represents the median expression level of the cluster. The green line represents the median expression level of the cluster after maternal *Yap1* knockout. (**E**) Venn diagrams showing the overlap of Z-decay transcripts (FPKM (GV) > 2; FPKM (zygote/GV) ≥ 1/2; FPKM (2-cell/zygote) <1/2) and the maternal transcripts that were significantly accumulated in four-cell embryos derived from *Yap1^–/–^* oocytes (FPKM (4-cell/GV) < 1/2 in WT; FPKM (*Yap1^–/–^*/WT) >1 at the 4-cell stage). *P* = 1e–308 by two-tailed Student's *t*-test. (**F**): Venn diagrams showing the overlap of ZGA-dependent Z-decay transcripts (FPKM (GV) > 2; FPKM (zygote/GV) ≥ 1/2; FPKM (two-cell/zygote) < 1/2; FPKM (two-cell/zygote) ≥ 1/2 after α-amanitin treatment) and the maternal YAP1-dependent Z-decay transcripts, i.e. the overlapping transcripts in (**E**). *P* = 1e–548 by two-tailed Student's *t*-test. (G–H) RT-qPCR results showing the relative mRNA levels of zygotic transcripts (**G**) and Z-decay transcripts (**H**) in two-cell embryos overexpressing a dominant negative form of TEAD4 (FLAG-TEAD4^ΔTEA^) by mRNA microinjection at the zygote stage. Error bars, s.e.m. *P < 0.05, **P < 0.01 and ***P < 0.001 by two-tailed Student's *t*-test. *n* = 3 biological replicates. (**I**) Illustration showing a putative TEAD-binding site (M-CAT element) of the mouse *T*u*t7* gene in mm9 genome. M-CAT element locates at 1025 bp upstream of *T*u*t7* transcription start site, from 59 925 533 to 59 925 541 on the chromosome 13.

In the following experiments, we aimed to identify the key factors involved in the Z-decay of mouse embryos. We focused on two proteins: (i) Yes-associated protein-1 (YAP1), which is a maternally and zygotically expressed transcriptional co-activator of the TEAD family of transcription factors; and (ii) TEAD4, which is zygotically expressed and is required for cell fate specification in preimplantation mouse embryos (27). We focused on YAP1/TEAD4 because our previous study has shown that maternal *Yap1*knock-out mouse embryos exhibit a ZGA defect: they have a prolonged two-cell stage and develop into the four-cell stage at a much slower pace compared to the wild-type embryos (28). Transcriptome analyses results indicate that maternal transcripts fail to be removed in two- to four-cell embryos derived from maternal *Yap1* knock-out mice. Therefore, we hypothesized that maternal YAP1 is a key ZGA factor required for the Z-decay of maternal transcripts. Quantitative RT-PCR showed that α-amanitin slightly affected zygotic *Yap1* expression at the two-cell stage (Figure 2A). The relatively small decrease in *Yap1* expression upon inhibition of transcription is consistent with a previous conclusion that maternal YAP1 plays a more important role than zygotic YAP1 in the MZT (28). In untreated embryos, the expression level of *Tead4* was low from the GV to the zygote stage and *Tead4* increased >25-fold at the two-cell stage. This zygotic expression of *Tead4* was blocked by α-amanitin treatment (Figure 2B).

To identify whether YAP1-TEAD4-directed zygotic transcriptional targets participated in Z-decay, we profiled the transcripts of GV oocytes and four-cell embryos derived from WT and *Yap1^fl/fl^*;*Gdf9-Cre* female mice (GSE74344) (28). Embryos at the four-cell stage instead of two-cell stage were used in this experiment because: (i) our results in Supplementary Figure S1 showed that maternal transcripts were more completely removed at the four-cell stage than at the two-cell stage and (ii) a large portion of maternal *Yap1*-knockout embryos (designated termed *Yap1^♀−^^/^^♂^^+^*) were able to pass through the two-cell stage with a slow developmental rate but then arrested at the four-cell stage. The oocyte transcriptome at the GV stage was not significantly affected by the *Yap1*-deletion (Figure 2C). However, the transcriptome of *Yap1^♀−^^/^^♂^^+^* embryos was significantly different from that of WT embryos at the four-cell stage: 1315 and 2117 transcripts were downregulated and upregulated in *Yap1^♀−^^/^^♂^^+^* embryos, respectively (Figure 2C). We have shown in the previous paper that the downregulated transcripts are zygotic transcription products (28). Strikingly, among the 4347 maternal transcripts being degraded in WT embryos during the GV to four-cell transition, 3197 were stabilized after maternal *Yap1*-deletion (Figure 2D). Gene set enrichment analysis of the maternal transcripts revealed that 1285 of YAP1-dependent degraded transcripts corresponded to recognized Z-decay transcripts (in Cluster II) (Figure 2E). Moreover, two thirds (784/1285) of YAP1-dependent Z-decay transcripts were also ZGA-dependent Z-decay transcripts in Cluster II (a) (Figure 2F).

To further assess the role of YAP1-TEAD4 in the Z-decay pathway, we overexpressed a dominant negative TEAD4 mutant, TEAD4^ΔTEA^, which lacks the TEA domain and cannot bind to DNA (29), in mouse zygotes by mRNA microinjection, and cultured these zygotes to the two-cell stage. Overexpression of TEAD4^ΔTEA^ blocked the transcriptional activation of *Rpl13* and *Rrm2*, two early zygotic genes that are directly regulated by maternal YAP1 (Figure 2G), indicating that TEAD4^ΔTEA^ indeed has an inhibitory effect on ZGA. On the other hand, some Z-decay transcripts were accumulated in embryos overexpressing TEAD4^ΔTEA^ (Figure 2H).

Recent studies have revealed that 3′-terminal uridylyl transferase 4 and 7 (TUT4/7)-dependent mRNA 3′-oligouridylation participate in mRNA decay and sculpts the mammalian maternal transcriptome (22,30). The promoter of the *Tut7* gene contains a putative TEAD-binding site at about 1000 bp upstream of its transcription start site (Figure 2I). Activation of zygotic *Tut4*/*7* in 2-cell embryos was also blocked by TEAD4^ΔTEA^ (Figure 2G). These results were consistent with those observed in *Yap1^♀−^^/^^♂^^+^* embryos and provided evidence that YAP1-TEAD4-mediated zygotic transcription, including *Tut4/7*, is upstream of Z-decay in mouse embryos.

### Terminal uridylyl transferases (TUTs) mediate Z-decay in mouse embryos

**Figure 3.**
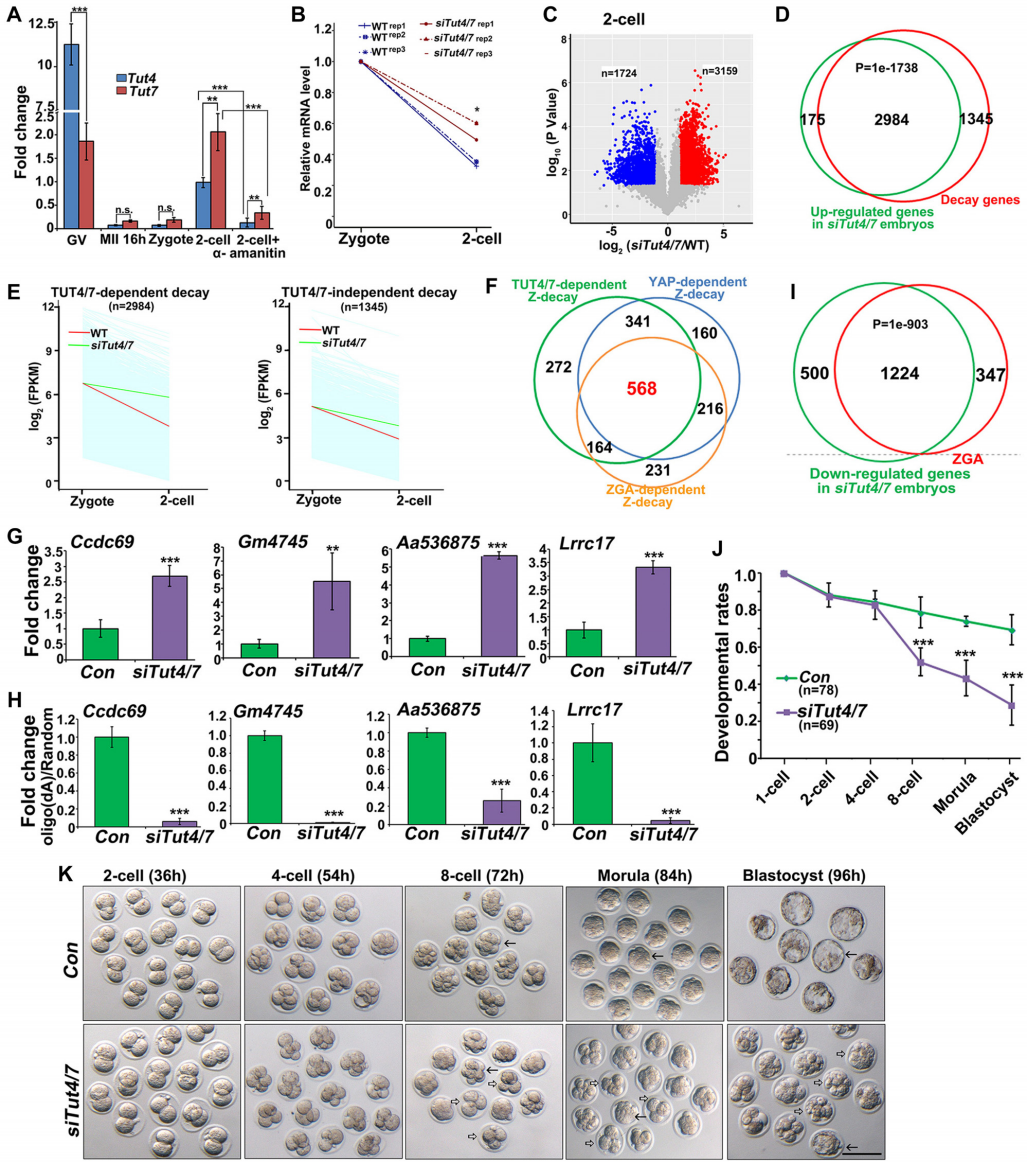
Role of zygotic TUT4 and TUT7 in the Z-decay pathway of mouse embryos. (**A**) RT-qPCR results showing the relative mRNA levels of indicated transcripts in mouse oocytes (GV and MII), zygotes, and two-cell embryos (with or without 25 ng/μl α-amanitin treatment). Error bars, s.e.m. **P < 0.01 and ***P < 0.001 by two-tailed Student's *t*-test. n.s.: non-significant. *n* = 3 biological replicates. (**B**) Changes of relative mRNA copy numbers in WT and *Tut4/7*-depleted embryos at the indicated stages. **P < 0.01 by two-tailed Student's *t*-test. (**C**) Scatter plot comparing transcripts between WT and *Tut4/7*-depleted two-cell embryos. Transcripts decreased or increased >2-fold in *Tut4/7*-depleted embryos were highlighted in blue or red, respectively. (**D**) Venn diagrams showing the overlap of upregulated transcripts in *Tut4/7*-depleted embryos (FPKM (*siTut4/7*/WT) > 2 in two-cell embryos) and the degraded transcripts from the zygote to 2-cell embryos in WT (FPKM (two-cell/zygote) < 1/3 in WT). *P* = 1e–1738 by two-tailed Student's *t*-test. (**E**) Degradation pattern of maternal transcripts in mouse embryos with or without zygotic *Tut4/7* depletion. Transcripts with FPKM (2-cell/zygote) < 1/3 were selected for the analyses. Each light blue line represents the expression level of one gene. The middle red line represents the median expression level of the cluster. The green line represents the median expression level of the cluster after zygotic *Tut4/7* depletion. (**F**) Venn diagrams showing the overlap of ZGA-dependent Z-decay transcripts (FPKM (GV) > 2; FPKM (zygote/GV) ≥ 1/2; FPKM (two-cell/zygote) < 1/2; FPKM (two-cell/zygote) ≥ 1/2 after α-amanitin treatment), YAP1-dependent Z-decay transcripts, and TUT4/7-dependent Z-decay transcripts. (**G**) RT-qPCR results showing the relative mRNA levels of indicated Z-decay transcripts in two-cell embryos with or without zygotic *Tut4/7*-depletion. Error bars, s.e.m. **P < 0.01 and ***P < 0.001 by two-tailed Student's *t*-test. *n* = 3 biological replicates. (**H**) Changes in RT-qPCR results obtained from oligo-(dA)-versus random primer-mediated RT reactions reflecting the 3′-oligouridylation levels of selected Z-decay transcripts in two-cell embryos with or without zygotic *Tut4/7*-depletion. Error bars, s.e.m. ***P < 0.001 by two-tailed Student's *t*-test. *n* = 3 biological replicates. (**I**) Venn diagrams showing the overlap of downregulated transcripts in *Tut4/7*-depleted embryos (FPKM (*siTut4/7*/WT) > 2 in two-cell embryos) and the zygotically activated transcripts in WT embryos (FPKM (2-cell/zygote) > 2 in WT). *P* = 1e–903 by two-tailed Student's *t*-test. (J, K): Developmental rates (**J**) and representative images (**K**) of preimplantation embryos after zygotic *Tut4/7*-depletion. Time after hCG injection (h) and numbers of analyzed embryos are indicated (n). Error bars, s.e.m. **P < 0.01; ***P < 0.001 by two-tailed Student's *t*-test. n.s.: non-significant. Scale bar, 100 μm. Arrows and hollow arrows indicate the normal and arrested embryos, respectively. *n* = 3 biological replicates.

The loss of *Tut4/7* in growing oocytes results in abnormal accumulation of maternal transcripts and impaired meiotic maturation, thus preventing an evaluation of the direct function of TUT4/7 in mRNA decay during MZT (22). RT-qPCR showed that *Tut4* and *Tut7* are expressed at the GV stage, but their transcripts are almost completely removed during oocyte maturation (Figure 3A). Zygotic *Tut4/7* mRNAs were re-expressed as early as the two-cell stage. While *Tut4* mRNA levels were significantly higher than those of *Tut7* at the GV stage, the mRNA level of *Tut7* was 2-fold more abundant than that of *Tut4* in two-cell embryos. α-amanitin blocked the transcriptional activation of *Tut4/7* in two-cell embryos (Figure 3A). Consistent with this observation, a recent paper showed that transcript uridylation reaches highest levels at the two-cell stage in mouse embryos (31), coinciding with the time of zygotic *Tut4/7* expression.

Based on these results, we hypothesized that TUT4/7-dependent 3′-oligouridylation might be involved in Z-decay. We, therefore, depleted zygotic *Tut4/7* transcripts by small-interfering-RNA (siRNA) microinjection into mouse zygotes. The *Tut4/7* mRNA levels in siRNA-mediated *Tut4/7*-depleted embryos decreased to 20% of the controls (Supplementary Figure S3A). We simultaneously depleted *Tut4/7* after fertilization, and cultured embryos to the two-cell stage for RNA-seq. Gene expression levels were assessed as FPKM, and the relative mRNA copy number was evaluated using the External RNA Controls Consortium (ERCC) spike-in. All samples were analyzed in triplicate and showed high correlations (average *r_s_* = 0.945; Supplementary Figure S3B). The overall transcript abundance increased in *Tut4/7*-depleted two-cell embryos (Figure 3B). Specifically, 3159 and 1724 transcripts were up- and down-regulated regulated in *Tut4/7*-depleted embryos, respectively (Figure 3C). Gene set enrichment analysis of the transcripts revealed that 2984 of the 3159 upregulated transcripts were those being degraded after fertilization in WT embryos (Figure 3D). Among the 4329 Z-decay transcripts detected in this experiment, 2984 (68.93%) were stabilized in *Tut4/7*-depleted embryos (Figure 3E). Moreover, 1345 of TUT4/7-dependent Z-decay genes were also identified in previous RNA-seq experiments (GSE71434) as Z-decay transcripts (Supplementary Figure S3C). Among these, over two-thirds (909) overlapped with the YAP1-dependent Z-decay transcripts (Supplementary Figure S3D). More importantly, 568 out of 909 (62.48%) transcripts belonged to the previously identified ZGA-dependent Z-decay transcripts (GSE71434) (Figure 3F). RT-qPCR verified that several Z-decay mRNAs accumulated at the two-cell stage after *Tut4/7* depletion (Figure 3G). In comparison, individual depletion of *Tut4* or *Tut7* had a more limited effect on the Z-decay of representative transcripts (Supplementary Figure S3E), indicating that *Tut4* and *Tut7* have overlapping functions in this process. Together, these results demonstrate that YAP1-TEAD4-mediated zygotic *Tut4/7* expression is essential for Z-decay in mouse embryos.

We also used a published method to quantify the 3′-oligouridylation levels of maternal transcripts (32). Briefly, we reverse-transcribed the mRNAs of two-cell embryos using oligo-dA (12) primers, which have a preference for 3′-oligouridylated mRNAs (32). Meanwhile, the total transcripts of two-cell embryos were reverse-transcribed using random hexamer primers. Therefore, the ratio changes in RT-qPCR results obtained from the oligo-dA-mediated versus random-primer-mediated RT products reflect the 3′-oligouridylation levels of the given transcripts (Supplementary Figure S3F). Simultaneous depletion of *Tut4* and *Tut7* resulted in reduced 3′-oligouridylation of the Z-decay mRNAs (Figure 3H). These results indicate that TUT4 and TUT7 are involved in the 3′-terminal oligouridylation of Z-decay mRNAs.

Strikingly, not only was a large subset of the Z-dependent maternal transcripts upregulated in si*Tut4/7* embryos but, in addition, over 2000 transcripts were downregulated. Gene set enrichment analysis of the 1724 downregulated transcripts in *Tut4/7*-depleted embryos revealed that 1224 (70.99%) belonged to early zygotically activated genes in WT embryos (Figure 3I and Supplementary Figure S3G). Thus, ZGA is impaired upon *Tut4/7* depletion. Moreover, the majority of zygotic *Tut4/7*-depleted embryos failed to develop into blastocysts and were arrested at the 4–8-cell stages (Figure 3J and K).

Together, our data show that (1) 3′-oligouridylation of maternal mRNAs by TUT4/7 is a key mechanism of Z-decay, and (2) clearance of these maternal mRNAs is essential for ZGA and preimplantation embryo development.

### Maternal BTG4 functions during Z-decay in mouse embryos

**Figure 4.**
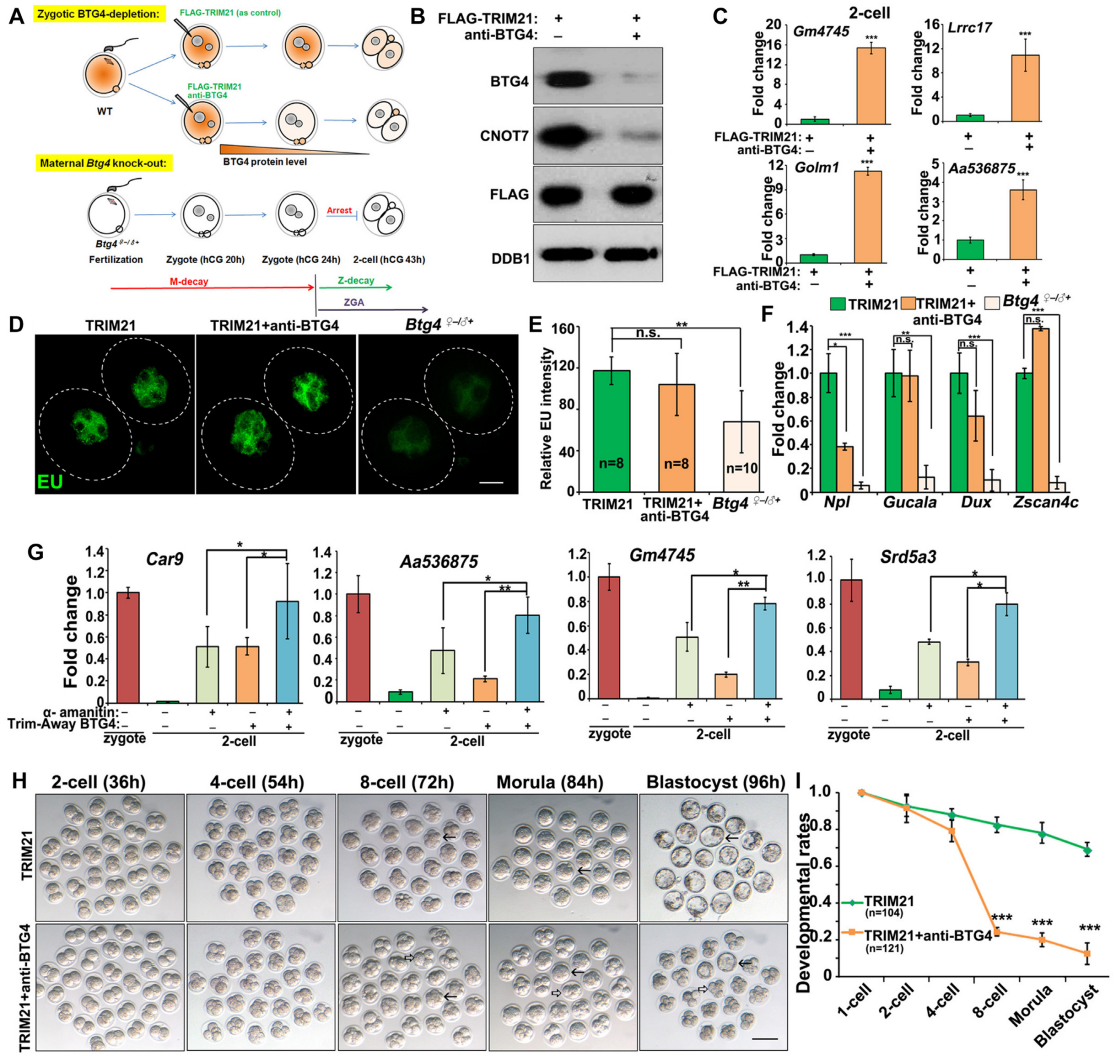
Dynamics of 3′-terminal polyadenylation and oligouridylation in maternal transcripts undergoing M-decay and Z-decay. (**A**) Illustration of BTG4 Trim-Away experiments. Zygotes were collected from *in vivo* at 20 h after hCG injection and were co-injected with *Flag-Trim21* mRNA and anti-BTG4 antibody or were only injected with *Flag-Trim21* mRNA as a negative control. Microinjected zygotes were cultured for another 4 h before sample collection for western blotting. (**B**) Western blot results showing the levels of BTG4, CNOT7, and TRIM21. Total proteins were collected from 100 zygotes at 4 h after microinjection and were loaded in each lane. DDB1 was blotted as a loading control. (**C**) RT-qPCR results showing the relative mRNA levels of indicated Z-decay transcripts in two-cell embryos with or without BTG4 Trimming-away. Error bars, s.e.m. ***P < 0.001 by two-tailed Student's *t*-test. *n* = 3 biological replicates. (D and E) Immunofluorescence (**D**) and quantification (**E**) of 5-ethynyl uridine (EU) fluorescence showing RNA transcription in two-cell embryos with or without BTG4 Trimming-away, and in maternal *Btg4* knockout two-cell embryos. Scale bar, 20 μm. Error bars, s.e.m. n.s.: non-significant. **P < 0.01 by two-tailed Student's *t*-test. (**F**) RT-qPCR results showing the relative mRNA levels of indicated zygotic transcripts in two-cell embryos with or without BTG4 Trimming-away. Error bars, s.e.m. n.s.: non-significant. *P < 0.05 and ***P < 0.001 by two-tailed Student's *t*-test. *n* = 3 biological replicates. (**G**) RT-qPCR results showing the relative mRNA levels of indicated transcripts in zygote and two-cell embryos with or without BTG4 Trim-away and α-amanitin treatment. Error bars, s.e.m. n.s.: non-significant. *P < 0.05 and ***P < 0.001 by two-tailed Student's *t*-test. *n* = 3 biological replicates. (**H-I**): Developmental rates (**H**) and Representative images (**I**) of preimplantation embryos after zygotic BTG4-depletion. Time after hCG injection (h) and numbers of analyzed embryos are indicated (n). Zygotes were microinjected as in (A), and then cultured until 96 h after hCG injection. Error bars, s.e.m. ***P < 0.001 by two-tailed Student's *t*-test. Scale bar, 100 μm. Arrows and hollow arrows indicate the normal embryos and arrested embryos, respectively.

It has been reported that the poly(A) tails of mRNAs need to be shortened to ∼25 bp through a CCR4-NOT-dependent mechanism before being oligouridylated by TUT4/7 (22,33). We previously showed that BTG4 recruits the CCR4-NOT deadenylase to translated transcripts to induce M-decay. Here, we investigated whether BTG4 and CCR4-NOT also play a role in the Z-decay process. Because maternal BTG4 proteins are present until the two-cell stage (17), we used a Trim-away technique (24,25) to induce rapid degradation of maternal BTG4 in zygotes (Figure 4A). Western blots showed that this treatment not only induced effective BTG4 degradation but also significantly reduced the level of CNOT7, the BTG4-binding subunit of the CCR4-NOT complex (Figure 4B). BTG4 and CNOT7 Trim-away resulted in failure to clear several Z-decay transcripts in two-cell embryos, indicating that BTG4 and CCR4-NOT is likely to play a role in the Z-decay process (Figure 4C).

EU staining showed that the overall transcriptional activation in two-cell embryos was impaired by maternal *Btg4* knockout but not by BTG4 Trim-away in zygotes (Figure 4D and E). RT-qPCR indicated that transcription of early zygotic genes at the two-cell stage was repressed by maternal *Btg4* knockout but was not affected or was only modestly affected by BTG4 Trim-away after fertilization (Figure 4F). α-Amanitin treatment or BTG4 Trim-away partially inhibited but not completely blocked the degradation of Z-decay transcripts (Figure 4G). In contrast, when we inhibited ZGA and depleted maternal BTG4 simultaneously, Z-decay of maternal transcripts was effectively abolished (Figure 4G). These results indicated that maternal BTG4 continues to mediate maternal mRNA clearance after fertilization, and participates in the Z-decay process together with ZGA-dependent factors.

Previous studies have demonstrated that maternal *Btg4* knockout impairs M-decay and causes arrest at the one- or two-cell stage (17). However, the zygotes with BTG4 trimmed-away developed beyond the two-cell stage but then arrested at the 4–8-cell stage (Figure 4H and I). Collectively, our results indicate that TUT4/7, BTG4, and the CCR4-NOT-deadenylase mediate Z-decay, which is crucial for the preimplantation development of mouse embryos.

### Poly(A) tail shortening and increased frequency of uridylation contribute to M-decay during oocyte maturation

**Figure 5.**
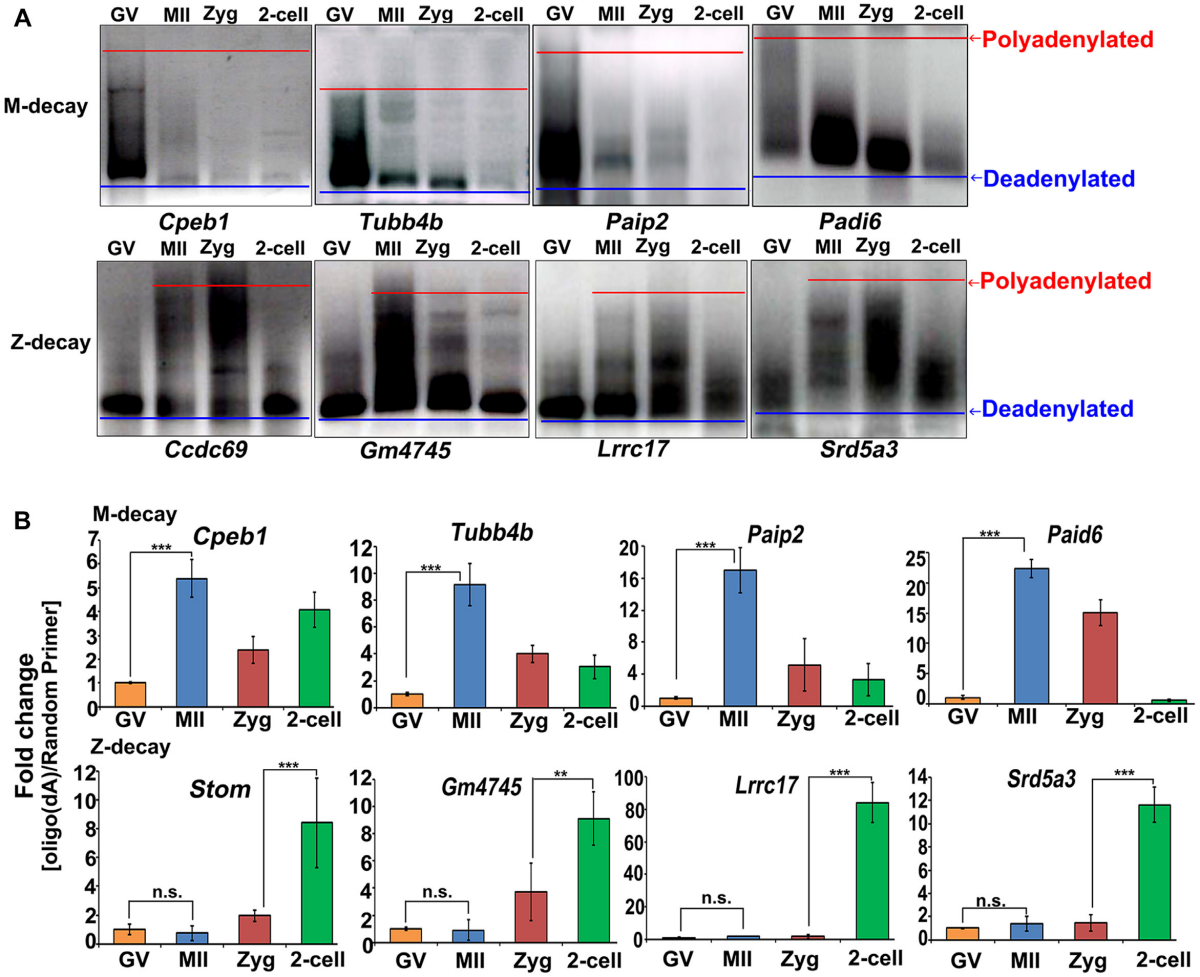
Dynamics of 3′-terminal polyadenylation and oligouridylation in maternal transcripts undergoing M-decay and Z-decay. (**A**) Poly(A) tail assay results showing changes in the poly(A)-tail length tendency of indicated M-decay and Z-decay transcripts during MZT. Zyg, zygote. Experiments were performed three times with reproducible results; a representative result is shown. (**B**) Changes in RT-qPCR results obtained with oligo-(dA)- versus random primer-mediated RT reactions reflecting the 3′-oligouridylation levels of selected M-decay and Z-decay transcripts during MZT. Zyg, zygote. Error bars, s.e.m. ***P < 0.001 by two-tailed Student's *t*-test. n.s.: non-significant. *n* = 3 biological replicates.

We next examined the factors that direct maternal mRNAs into the M-decay or Z-decay pathways. In cytoplasmic mRNA turnover, deadenylation of poly(A) tails is the initial and rate-limiting step (34). Results of the PAT assay showed that M-decay mRNAs (*Cpeb1*, *Tubb4b*, *Paip2* and *Padi6*) had long poly(A) tails in GV stage-arrested oocytes, but that their poly(A) tails rapidly shortened after meiotic resumption (Figure 5A, upper panels; Supplementary Figure S4A). In contrast, Z-decay mRNAs (*Ccdc69*, *Gm4745*, *Lrrc17* and *Srd5a3*) had short poly(A) tails in GV oocytes, were polyadenylated in maturing oocytes and in zygotes, followed by deadenylation at the two-cell stage (Figure 5A, lower panels; Supplementary Figure S4A). Closely related to the changes in poly(A) tail length, mRNA 3′-oligouridylation sculpted the mammalian maternal transcriptome, including both M-decay and Z-decay mRNAs. Oligo-(dA)-mediated RT efficacy of M-decay transcripts was remarkably increased during the GV-MII transition (Figure 5B, upper panels). This observation indicates that 3′-oligouridylation of M-decay transcripts occurs during the GV-MII transition. In contrast, the 3′-oligouridylation levels of Z-decay transcripts remained low from the GV to zygote stages, and significantly increased at the two-cell stage (Figure 5B, lower panels). We also performed 3′-ligation RACE analysis of *Gm4775* and *Lrrc17* transcripts in zygotes and early two-cell embryos. The RACE analysis showed that the occurrences of the terminal uridylation increased upon transition to the two-cell stage, and some transcripts in two-cell embryos have longer oligo(U) tails than those in zygotes (Supplementary Figure S4B). These results provide further evidence for the involvement of TUT4/7 in Z-decay, and suggest that selective 3′-oligouridylation at different stages of MZT contributes to distinct degradation patterns of M-decay and Z-decay mRNAs.

### A long 3′-UTR and active translation confer resistance of Z-decay transcripts to M-decay

**Figure 6.**
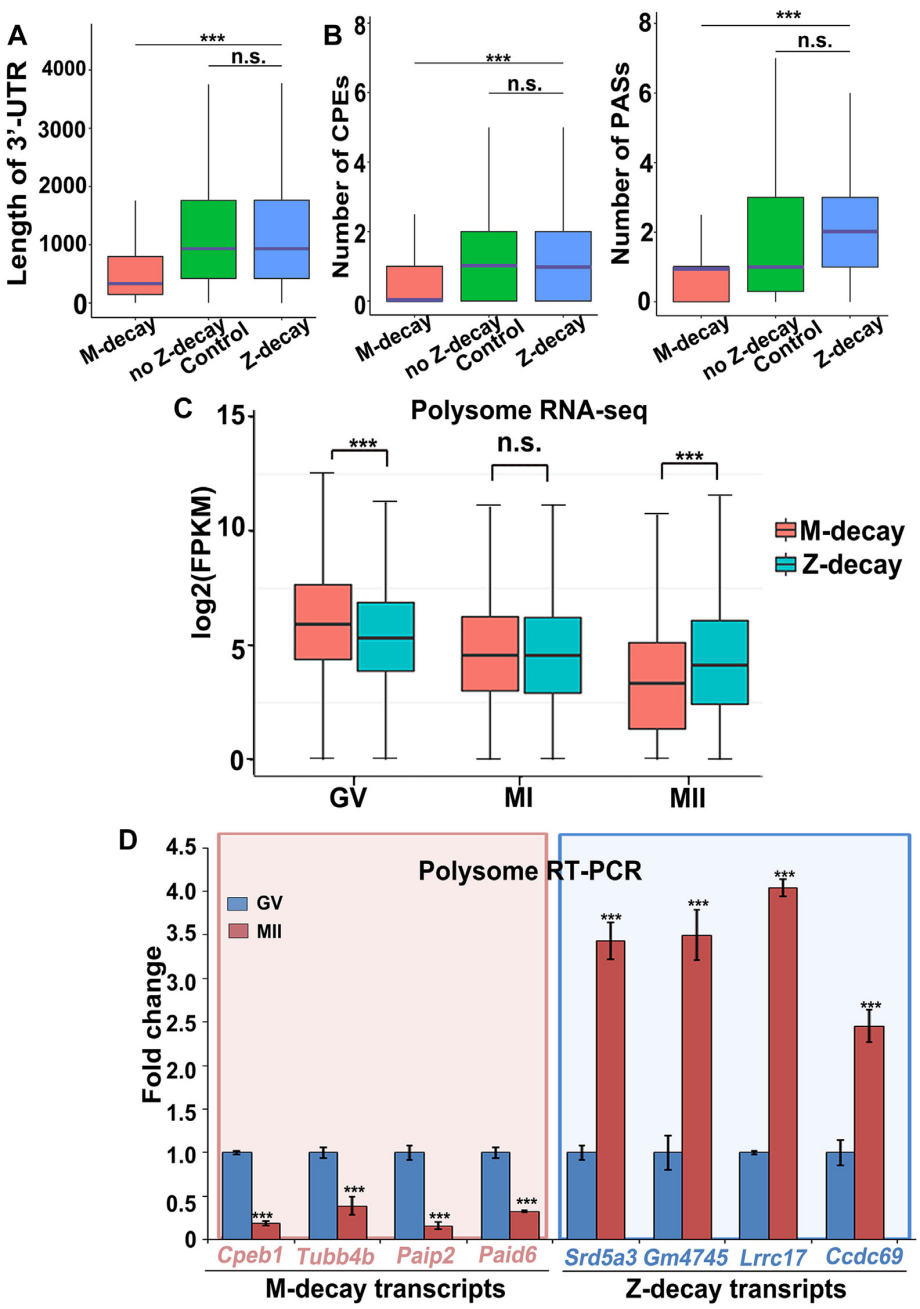
Influence of 3′-UTR length and translational activity on the timing of mRNA degradation. (A and B) Average 3′-UTR length (**A**) and numbers of CPEs and PASs (**B**) in the 3′-UTR of murine M-decay, Z-decay, and length-controlled non-Z decay transcripts. The box indicates upper and lower quantiles, the purple thick line in the box indicates the median.***P < 0.001 by two-tailed Student's *t*-test. n.s.: non-significant. (**C**) Levels of polysome-bound M-decay and Z-decay transcripts in oocytes at the GV, MI and MII stages (two biological repeats). The expression level of each transcript was normalized by the mCherry spike-in, which was *in vitro* transcribed and equally added to each sample before RNA extraction. The box indicates upper and lower quantiles, and the line in the box indicates the median. ***P < 0.001 by two-tailed Student's *t*-test. n.s.: non-significant. (**D**) RT-qPCR results showing the relative mRNA levels of M-decay and Z-decay transcripts in association with polysomes at the GV and MII stages. Error bars, s.e.m. ***P < 0.001 by two-tailed Student's *t*-test. *n* = 3 biological replicates.

In addition to the poly(A) tail, length of the 3′-UTR is also a factor that determines mRNA stability during MZT in zebrafish (35,36). By analyzing the RNA-sequencing results in mouse, we found that M-decay transcripts possessed shorter 3′-UTRs compared to Z-decay transcripts (Figure 6A). Cytoplasmic polyadenylation is controlled by cis-elements in the 3′-UTRs of mRNAs including the polyadenylation signal (PAS) and the cytoplasmic polyadenylation element (CPE) (37,38). When multiple CPEs and PASs are present in the 3′-UTR of mRNAs, they contribute to mRNA translation in an additive manner during oocyte maturation (39). More CPEs and PASs were present in the 3′-UTRs of Z-decay mRNAs than those in the 3′-UTRs of M-decay mRNAs (Figure 6B). Using UTR-length-controlled, non-Z decay genes as control, the results showed that the increased number of CPEs and PASs of Z-decay genes mainly depend on longer 3′-UTR (Figure 6B). These observations suggest that long 3′-UTRs and high translational activity of Z-decay mRNAs may confer resistance to CCR4-NOT-mediated deadenylation.

To provide direct evidence for whether Z-decay mRNAs are actively translated during oocyte maturation, we analyzed the RNA-sequencing data of polyribosome-bound mRNAs at GV, MI and MII stages. Although zygote samples were not included in this experiment, which was performed previously (GSE118564 (20)), the profiles of maternal mRNAs in MII oocytes can be used for the purpose of this study. At the GV stage, significantly more M-decay mRNAs than Z-decay mRNAs co-fractionated with polyribosomes (Figure 6C). In contrast, significantly more Z-decay mRNAs than M-decay mRNAs co-fractionated with polyribosomes at the MII stage (Figure 6C). Therefore, more Z-decay than M-decay transcripts participated in translation during oocyte maturation, especially at the MII stage. Using RT-qPCR, we verified the RNA-seq results for several transcripts and demonstrated increased recruitment of selective Z-decay transcripts to polyribosomes for active translation during the GV-MII transition (Figure 6D). In contrast, the binding of M-decay transcripts to polyribosomes was decreased during meiotic maturation (Figure 6D). Taken together, Z-decay mRNAs were more actively polyadenylated and translated compared to M-decay mRNAs after meiotic resumption. As a consequence, they were more resistant to CCR4-NOT-mediated deadenylation and TUT4/7-dependent-oligouridylation compared to M-decay transcripts during oocyte maturation.

## DISCUSSION

Turnover of maternal transcripts was first reported in mouse oocytes >20 years ago (40,41); however, little was known regarding the degradation machinery and mechanisms of specific transcript targeting until recently (1). Here, we have shown that both maternal and zygotic transcript degradation pathways function in the early mouse embryo during MZT. The sequential actions of both pathways are necessary for timely elimination of subgroups of maternal transcripts, and coordinate crucial developmental events during the MZT.

**Figure 7.**
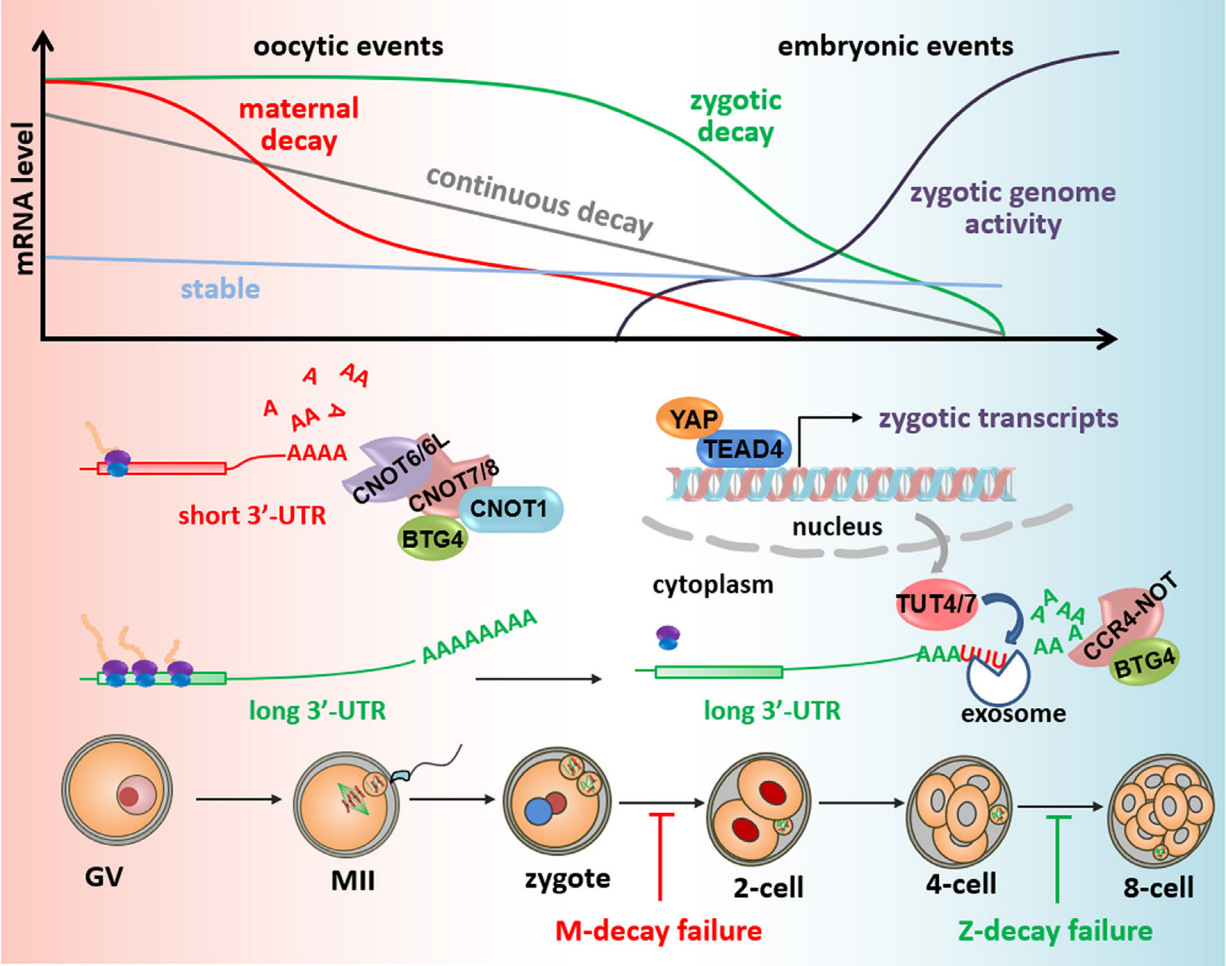
A summary of maternal mRNA clearance during mouse oocyte maturation and MZT. Maternal mRNAs can be classified into four categories based on their degradation dynamics: maternal decay (M-decay), zygotic decay (Z-decay), continuous decay, and stable throughout the MZT process. The maternal processes are in the pink area and the zygotic events are in the blue area. M-decay transcripts usually have short 3′-UTRs and are deadenylated by the CCR4-NOT complex during oocyte maturation. In contrast, Z-decay transcripts tend to have long 3′-UTRs, maintain long poly(A) tails, bind with polysomes, and are actively translated in maturing oocytes. After ZGA, maternally provided YAP1, together with zygotically expressed TEAD4, induce expression of zygotic TUT4/7, which mediate the 3′-oligouridylation and degradation of Z-decay transcripts. In addition, maternally translated BTG4 and CNOT7 continue to be required for Z-decay. Their transcripts are among those being removed as late as the four-cell stage. Blockage of Z-decay by depleting TUT4/7 or BTG4 in zygotes caused developmental arrest of embryos at the eight-cell stage, indicating that Z-decay is an essential MZT event.

Previous studies have demonstrated the existence of ZGA-dependent maternal mRNA decay in mice (14,42,43), but did not describe this in detail. In this study, we investigated the mechanisms that mediate Z-decay as summarized in Figure 7. These mechanisms show both similarities and differences between mammals and other model systems. In zebrafish, codon usage and 3′-UTR length determine maternal mRNA stability during the MZT (35,36). We observed a similar trend in mouse: at the whole transcriptome level, the transcripts destined for Z-decay have longer 3′-UTRs, and consequently have more cis-regulatory elements, than those of M-decay transcripts. In maturing oocytes, these Z-decay transcripts were highly polyadenylated and bound by polyribosomes, both reflecting active translation. Active translation may inhibit the Z-decay transcripts from being targeted for degradation during oocyte maturation. After ZGA, a transient increase in TUT4/7-mediated 3′-oligouridylation would facilitate the clearance of these M-decay-resistant mRNAs.

Previous studies have shown that M-decay is not a single pathway. Svoboda *et al.* have outlined four specific phases of transcript degradation during oocyte development and MZT (44). Among these M-decay involves first three distinct phases, while the Z-decay pathway corresponds to the last phase. The three phases of M-decay include: (i) gradual transcript clearance (also called transcriptome sculpting) during oocyte growth. These transcripts are targeted by endogenous RNA inference pathway or *Tut4/7*-guided exosomes during oocyte growth (22); (ii) transcripts whose degradation is triggered or accelerated after meiotic resumption in fully grown oocytes. These are transcripts whose degradation depends on 3′-tail shortening by CCR4-NOT deadenylase (20) and 5′-end decapping (45), and is largely accomplished by the MII stage. Many of these transcripts encode ‘housekeeping’ proteins such as ribosomal components (Cluster 1 in Figure 1B) (46); (iii) transcripts relatively stable until ovulation whose degradation is accelerated upon fertilization (Cluster 2 in Figure 1B). Mechanistically, these transcripts may be targeted by CCR4-NOT with the help of its adaptor BTG4 (which only start to accumulate after meiotic resumption) (17,39) or by some other inadequately investigated deadenylases. Recognition of these different components of M-decay is important for accurate interpretation of phenotypes of different knockout mice and functions of proteins encoded by these genes.

Unlike in zebrafish, zygotic miRNAs do not contribute to MZT in mouse (6,47). Instead, early zygotic expression of *Tead4* and *Tut4/7* is required for Z-decay in mouse. Zygotically expressed TEAD4, together with its maternally provided cofactor, YAP1, mediate zygotic expression of *Tut4/7* genes that encode the potential key effectors of Z-decay. Maternal transcripts destined to be removed by the Z-decay pathway, accumulate in 4-cell embryos derived from oocyte-specific *Yap1* knockout mice.

The involvement of TUT4/7-mediated 3′-oligouridylation in maternal mRNA decay during MZT has been reported in zebrafish and *Drosophila* (31). A transient increase in mRNA 3′-oligouridylation was also observed in mouse embryos at the two-cell stage ((31) and this study). However, knockout of *Tut4/7* in developing oocytes severely disturbed the maternal transcriptome and caused oocyte maturation failure (22). These phenotypes prevented evaluation of a possible direct function for TUT4/7 in the MZT. We have shown that maternal *Tut4/7* mRNAs are removed by the M-decay pathway and then re-expressed as early zygotic transcripts in both mice and humans. The temporal correlations among zygotic *Tut4/7* expression, maternal mRNA 3′-oligouridylation, and Z-decay encouraged us to hypothesize that zygotic TUT4/7-mediated 3′-oligouridylation facilitates Z-decay (Figure 7).

Since there were no spike-in controls in the RNA-seq data that we used to define the four clusters (26), we did not perform any specialized normalization steps to eliminate the biases of RNA degradation. In this condition, the transcripts in cluster IV are transcripts with average stability from the GV stage to late two-cell stage, whereas the transcripts in cluster I and cluster II are those that preferentially degrade from the GV to zygote and from the zygote to late two-cell stage, respectively. However, qPCR data can reflect the absolute quantity changes of RNA, and this could account for the difference between RNA-seq and qPCR data in Supplementary Figure S1B (GV versus two-cell).

In all these model systems, there is no distinct division of labor between M-decay and Z-decay during MZT. When Z-decay is inhibited by transcriptional inhibitors, the M-decay pathway still mediates the degradation of Z-decay transcripts, albeit at a much slower rate. The same phenomenon was also observed in *Drosophila*, *Xenopus* and zebrafish embryos (5,6). The components of the M-decay pathway, such as BTG4 and CCR4-NOT complex, continue to function in Z-decay, but they need reinforcements from zygotic factors, such as microRNAs in zebrafish and TUT4/7 in mouse, for the timely removal of stable maternal mRNA species.

Despite the fact that the M-decay pathway can mediate slow degradation of Z-decay transcripts when given enough time under experimental conditions, the Z-decay pathway is physiologically essential for early embryo development, because embryonic development cannot be delayed. Maternal transcripts, particularly those functioning in meiosis, need to be degraded in a timely manner to allow smooth mitotic cell cycle progression. Our results demonstrate a tight link between maternal mRNA decay and mitosis in early embryos. When M-decay is impaired in embryos derived from *Btg4* null female mice, zygotes arrest at the one- to two-cell stage (17,20). In comparison, when Z-decay is blocked by BTG4 trim-away or zygotic *Tut4/7*-depletion, most embryos complete the first two mitotic cycles but arrest at the four-cell stage. These results suggest that M-decay and Z-decay are prerequisites for the first two and the third mitosis cycles, respectively.

## DATA AVAILABILITY

RNA-seq data have been deposited in the NCBI Gene Expression Omnibus database under accession code GSE128283.

## Supplementary Material

gkz1111_Supplemental_File
