# Supplementary material for: Characterization of zygotic genome activation-dependent maternal mRNA clearance in mouse
Source: Nucleic Acids Res. 2019 Nov 28;48(2):879–94. doi: 10.1093/nar/gkz1111 (PMC6954448; doi:10.1093/nar/gkz1111)
Supplement: gkz1111_Supplemental_File [file gkz1111_supplemental_file.pdf]

Characterization of Zygotic Genome Activation-dependent Maternal mRNA Clearance in Mouse

Qian-Qian Sha[1,2#], Ye-Zhang Zhu[1#], Sen Li[2], Yu Jiang[1], Lu Chen[1], Xiao-Hong Sun[2], Li Shen[1*], Xiang-Hong Ou[2*], Heng-Yu Fan[1,3*]

[1]MOE Key Laboratory for Biosystems Homeostasis & Protection and Innovation Center for Cell Signaling Network, Life Sciences Institute, Zhejiang University, Hangzhou 310058, China
[2]Fertility Preservation Laboratory, Reproductive Medicine Center, Guangdong Second Provincial General Hospital, Guangzhou 510317, China
[3]Key Laboratory of Reproductive Dysfunction Management of Zhejiang Province; Assisted Reproduction Unit, Department of Obstetrics and Gynecology, Sir Run Run Shaw Hospital, School of Medicine, Zhejiang University, Hangzhou 310016, China

Supplementary Information:

Supplementary Figure S1-S4
Supplementary Table S1-S4

Supplementary Figures

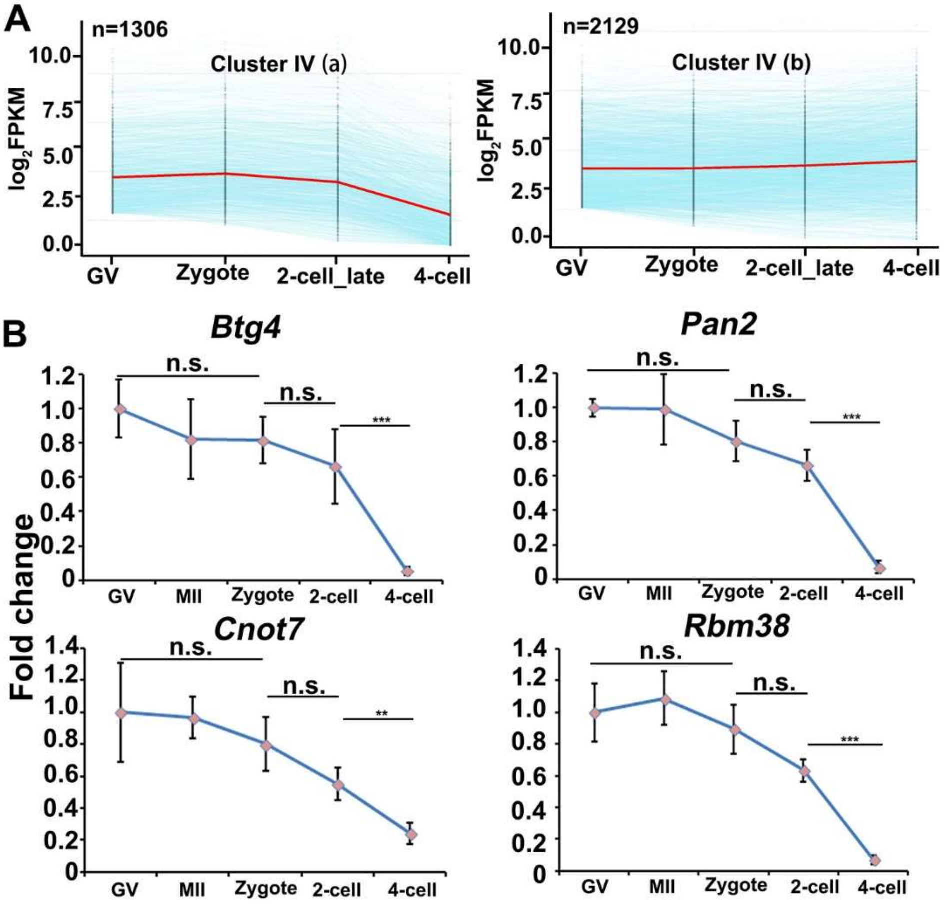

Figure S1: Dynamics of mouse cluster IV maternal transcripts from the GV to 4-cell stages. **A:** Expression pattern of mouse maternal transcripts from the GV to 4-cell stages. Transcripts with FPKM ≥ 1 at the GV stage and ≥ 1/2 at the 2-cell stage were selected. Each light blue line represents the expression level of one gene, and the middle red line represents the median expression level of the cluster. **B:** RT-qPCR results showing the relative mRNA levels of select transcripts in oocytes and embryos at the indicated developmental stages. Error bars, s.e.m. **: $P < 0.01$ and ***: $P < 0.001$ by two-tailed Student's *t*-test. n.s.: non-significant. n = 3 biological replicates.

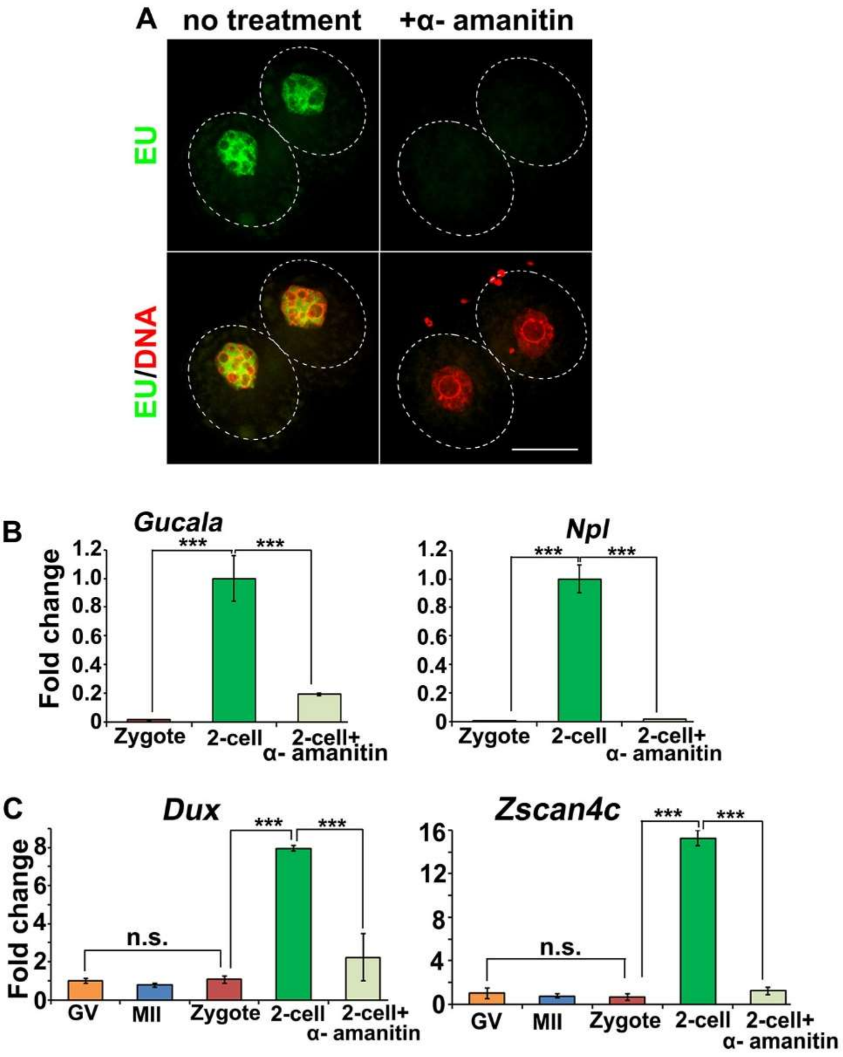

Figure S2: Effect of α-amanitin treatment on maternal mRNA decay in mouse zygotes.

A: 5-Ethynyl uridine (EU) fluorescence showing RNA transcription in 2-cell embryos with or without α-amanitin (25 ng/μl) treatment. The embryos were treated with α-amanitin as early as the zygote stage and cultured to the 2-cell stage. For each genotype, 10 embryos were observed with similar results. Scale bar, 50 μm. n = 3 biological replicates. **B:** qRT-PCR results showing the relative mRNA levels of indicated transcripts in zygotes and 2-cell embryos (with or without 25 ng/μl α-amanitin treatment). Error bars, s.e.m. ***: $P < 0.001$ by two-tailed Student's *t*-test. n = 3 biological replicates. **C:** RT-qPCR results showing the relative mRNA levels of indicated transcripts in mouse oocytes (GV and MII), zygotes and 2-cell embryos (with or without 25 ng/μl α-amanitin treatment). Error bars, s.e.m. ***: $P < 0.001$ by two-tailed student's *t*-test. n.s.: non-significant. n = 3 biological replicates.

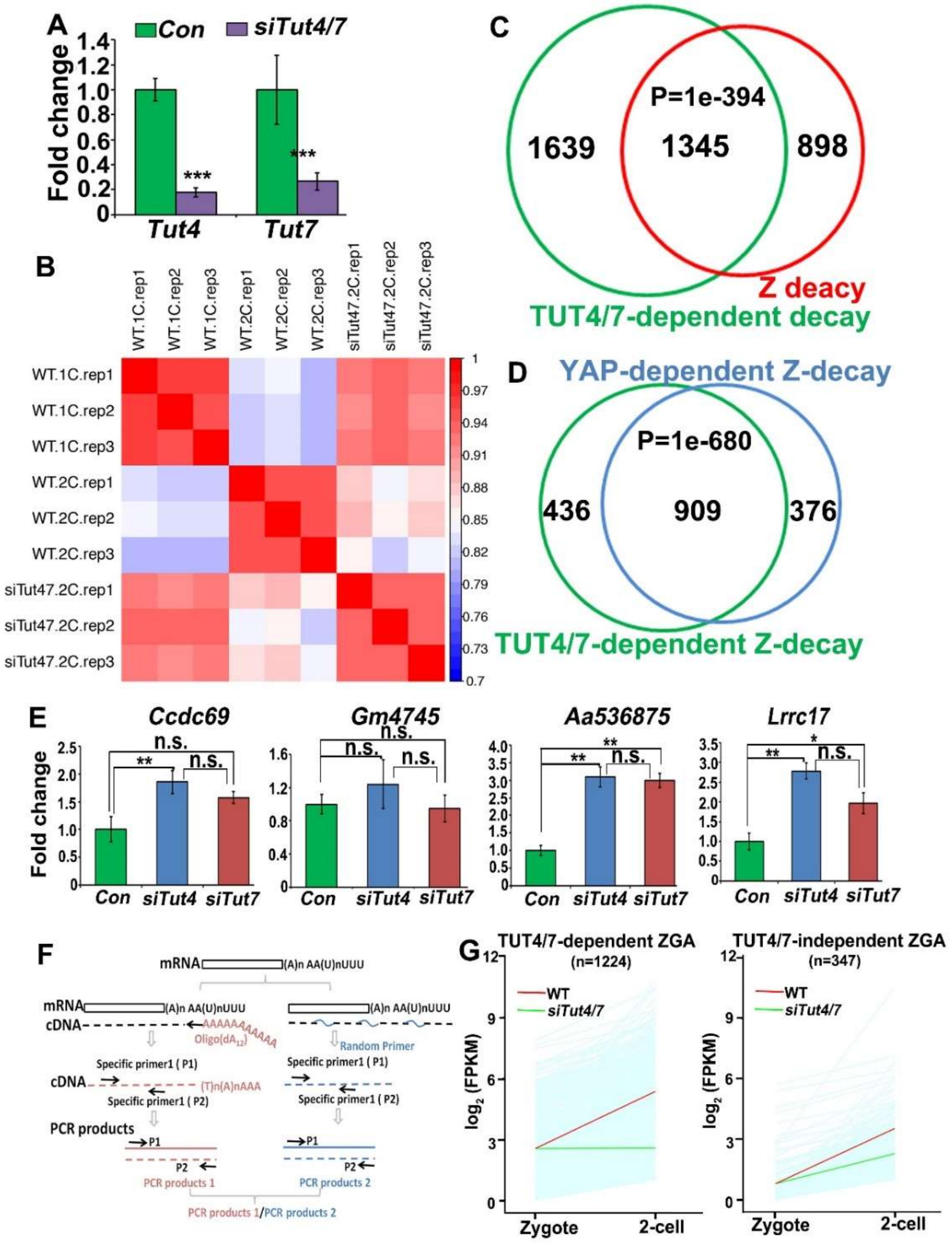

Figure S3: Role of maternal YAP1 and zygotic TUT4/7 in the Z-decay of maternal transcripts.

A: RT-qPCR results showing the RNAi depletion efficiency of *Tut4/7* in 2-cell embryos. Small-interfering-RNAs (siRNAs) were microinjected into zygotes at 24 h after hCG injection. n = 3 biological replicates. Error bars, s.e.m. ***: P < 0.001 by two-tailed Student's t-test. **B:** Heatmap of spearman correlation coefficients of total transcripts among WT and *siTut4/7* embryos at different stages. **C:** Venn diagrams showing the overlap of the Z-decay transcripts and TUT4/7-dependent decay transcripts. P = 1e-394 by two-tailed student's t-test. **D:** Venn diagrams showing the overlap of YAP1-dependent Z-decay transcripts and TUT4/7-dependent Z-decay transcripts. P=1e-680 by two-tailed student's t-test. **E:** RT-qPCR results showing the

relative mRNA levels of selected Z-decay transcripts in 2-cell embryos after *Tut4* or *Tut7* depletion. Error bars, s.e.m. *: $P < 0.05$ and **: $P < 0.01$ by two-tailed student's *t*-test. n.s.: non-significant. n = 3 biological replicates. **F:** Strategy of the mRNA poly(U) tail assay. The same pool of oocyte total RNAs was split and reverse transcribed using oligo-(dA) and random primers. P1 and P2, gene-specific primers. **G:** Activation pattern of maternal transcripts in mouse embryos with or without zygotic *Tut4/7* depletion. Transcripts with FPKM (2-cell/zygote) > 2 were selected for analyses. Each light blue line represents the expression level of one gene. The middle red line represents the median expression level of the cluster. The green line represents the median expression level of the cluster after zygotic *Tut4/7* depletion.

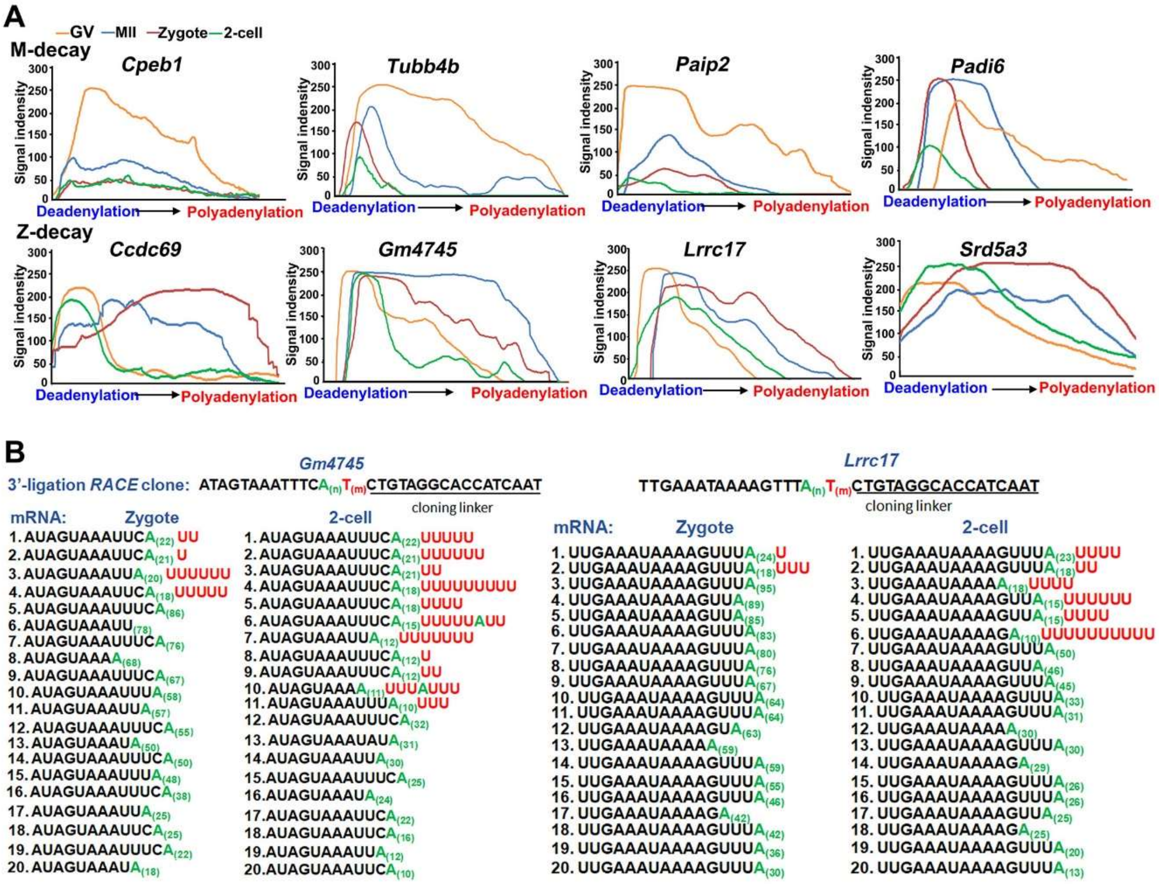

Figure S4: Detection of 3′-terminal polyadenylation and oligouridylation in maternal transcripts undergoing M-decay and Z-decay.

A: Quantification of the PAT assay results in Figure 5A. The plots show the averaged relative signal intensity (y axis) and the length of the PCR products based on mobility (x axis). **B:** Subcloning sequencing results of 3′-ligation RACE PCR products of *Gm4775* and *Lrrc17* transcripts at the zygote and the early 2-cell stages.

Supplementary Tables

Supplementary Table S1. Primer sequences

Primer name	Genes targeted	Application	Sequences (5’-3’)
Ccdc69-F	*Ccdc69*	Real-time qPCR	5’-CCCAAGAAGTGCCTTTCCTTA-3’
Ccdc69-R			5’-GCCACGGGAGAAGTCTTAAAT-3’
Gm4745-F	*Gm4745*	Real-time qPCR	5’-TGTGGAGCTGGCTGTATTG-3’
Gm4745-R			5’-GAGGTTCATCTGCCTGTACTTAG-3’
Aa536875-F	*Aa536875*	Real-time qPCR	5’-GTGATCTGTCTCTGGCTGAAAG-3’
Aa536875-R			5’-TGAGTGTTCCTGCATCCATTAG-3’
Car9-F	*Car9*	Real-time qPCR	5’-AGCCGCTACTACCGATATGA-3’
Car9-R			5’-CAAGGAAACGGAGAGAGTATGG-3’
Srd5a3-F	*Srd5a3*	Real-time qPCR	5’-GTCATCCGCCCATCAGTATAAA-3’
Srd5a3-R			5’-GTACTCGAACCAGTCTCCAAAG-3’
Rpl13-F	*Rpl13*	Real-time qPCR	5’-CGAGGAGGCGAAACAAGTC-3’
Rpl13-R			5’-TTCGGAGACTGGCAAAAGC-3’
Rrm2-F	*Rrm2*	Real-time qPCR	5’-AGTTCCTCACGGAGGCCTT-3’
Rrm2-R			5’-CTCTGATACTCGCCTACTCGC-3’
Tut4-F	*Tut4*	Real-time qPCR	5’-CCTCCAAAGCAAAATCCAGTG-3’
Tut4-R			5’-GAAGACAGAAACAGACAACCA-3’
Tut7-F	*Tut7*	Real-time qPCR	5’-ACCTGTGTTTCAGACCCTTAC-3’
Tut7-R			5’-CAGCTGCATTTCCCAGTTTATC-3’
Bcl210-F	*Bcl210*	Real-time qPCR	5’-CCACTGCATGAACGCACTAGA-3’
Bcl210-R			5’-GAGCAACTTATCTGCCATCTGT-3’
Rgs2-F	*Rgs2*	Real-time qPCR	5’-GAGAAAATGAAGCGGACACTCT-3’
Rgs2-R			5’-GCAGCCAGCCCATATTTACTG-3’
Lrrc17-F	*Lrrc17*	Real-time qPCR	5’-ACAGCATCCACTACCTCTACT-3’
Lrrc17-R			5’-CCACAGACCACCCTTTGTATT-3’
Cpeb1-F	*Cpeb1*	Real-time qPCR	5’-GGGTCTGGTCGTGTGACTTT-3’
Cpeb1-R			5’-TGGTGGTTTTGATCTCCACA-3’
Tubb4b-F	*Tubb4b*	Real-time qPCR	5’- CACTTACCACGGAGATAGCGA-3’
Tubb4b-R			5’- ACCTTCTGTGTAGTGCCCCTT-3’
Paip2-F	*Paip2*	Real-time qPCR	5’- GAACGCTGTTTCCAAGAAATGC-3’
Paip2-R			5’-CCAGAGAAGAGCCATCACTGATA-3’
Paid6-F	*Paid6*	Real-time qPCR	5’-AGTGTATCAGCCTGAACCGC-3’
Paid6-R			5’-AGGTGCCATTGATTTTGGG-3’
Stom-F	*Stom*	Real-time qPCR	5’-AGGGAGATTAGCCAGAAACTTG-3’
Stom-R			5’-GAGTCCCTCATCTGCCTTTATG-3’

Izumolr-F	*Izumolr*	Real-time qPCR	5'-CTTCTCTCTGTGCCTGTTGT-3'
Izumolr-R			5'-GAATAGCCTGAGGATGGAGATG-3'
Golm1-F	*Golm1*	Real-time qPCR	5'-CCTATGACCTGAACCAGTGTATC-3'
Golm1-R			5'-GCTTTCTGATGACCTCCTCTATC-3'
Gucala-F	*Gucala*	Real-time qPCR	5'-ATCTAGGGCATCCGTCTCTT-3'
Gucala-R			5'-GATGGGCACTCCGTCATAAA-3'
Npl-F	*Npl*	Real-time qPCR	5'-GGCCTTCCCTAAGAAGAAACTC-3'
Npl-R			5'-CCTGTTCCTTCACCAGGTAATC-3'
Dux-F	*Dux*	Real-time qPCR	5'-AGCGACTCAAACTCCTTCTTC-3'
Dux-R			5'-CTGTGCTGTCTGCTTGAGT-3'
Zscan4c-F	*Zscan4c*	Real-time qPCR	5'-GCCTTATGTCTGTTCCCTATGT-3'
Zscan4c-R			5'-CAGTCTCTGCTGAGGATGTTAG-3'
Btg4-F	*Btg4*	Real-time qPCR	5'-TGAAAAAGCATGAGAAACTGAGTAC-3'
Btg4-R			5'-CCCATCTACCTTTAAAAGAAGCAA-3'
Cnot7-F	*Cnot7*	Real-time qPCR	5'-GGTGGATTACAGGAAGTTGCTG-3'
Cnot7-R			5'-GGATGAGCCAGAACCAAGG-3'
Pan2-F	*Pan2*	Real-time qPCR	5'-TCCGGCCTTCTTACGATTCAT-3'
Pan2 -R			5'-GGCCTGTGGGTTCACAAAAC-3'
Rbm38-F	*Rbm38*	Real-time qPCR	5'-TGCTCCCCGAGTGTGTTTC-3'
Rbm38-R			5'-GTACTTTCTGAGCGATGCGTC-3'
Gapdh-F	*Gapdh*	Real-time qPCR	5'-ACACTGAGGACCAGGTTGTCTC-3'
Gapdh-R			5'-TACTCCTTGGAGGCCATGTAG-3'
Gfp-F	*Gfp*	Real-time qPCR	5'-CTCGTGACCACCCTGACCTA-3'
Gfp-R			5'-ATGCCCTTCAGCTCGATG-3'
Tead4-F	*Tead4*	Real-time qPCR	5'-GGAGTATGCCCGCTATGAGA-3'
Tead4-R			5'-TCCTGTGTGTCTCGGTTGGT-3'
P1	N.A.	dT anchor primer (P1)	5'-GCGAGCTCCGCGGCCGCGT12-3'
Cpeb1	*Cpeb1*	PAT assay (P2)	5'-ACAGTTGACAAAGACGCTACTGGAATTGAAAACTTGA-3'
Tubb4b	*Tubb4b*	PAT assay (P2)	5'-GAACGACCTGGTGTCCGAGTACCAGCAGTA-3'
Paip2	*Paip2*	PAT assay (P2)	5'- GTTAAGCTGCCATACGTGTTC-3'
Paid6	*Paid6*	PAT assay (P2)	5'- GGGAGTTAGGGTCTCTCACATC-3'
Ccdc69	*Ccdc69*	PAT assay (P2)	5'-GAGACACAATCCAAGAAACAGGAACACAGT-3'
Gm4745	*Gm4745*	PAT assay (P2)	5'-CTGAGAAACCCCTCCACTCCAGCTTACA-3'
Lrrc17	*Lrrc17*	PAT assay (P2)	5'-AGTACACAGTGGGCCAGAACTGCTGGT-3'

Srd5a3	*Srd5a3*	PAT assay (P2)	5’-TCCAGGGAGTAAAGAGTTTGTTGGAGTGAGT-3’
Tut4-F1	*Tut4*	siRNA	5’-CCAGCUCUCUGAAUAGAAATT-3’
Tut4-F2			5’-GGAGGAAAUGUCAAAGGUUTT-3’
Tut7-F1	*Tut7*	siRNA	5’-CCAGCAAUCCAUAUGCAUUTT-3’
Tut7-F2			5’-GCCUGCCACCAUAUGUGUUTT-3’

Supplementary Table S2. Antibody information

Protein name	Manufacture (catalogue number)	Applications (working dilution)	Website Link*
**CNOT7**	Abcam (ab195587)	WB (1:1000)	http://www.abcam.com/cnot7-antibody-epr18722-ab195587.html
**BTG4**	Abcam (ab206914)	WB (1:1000)	https://www.abcam.cn/btg4-antibody-eprzju-21-ab206914.html
**FLAG**	Sigma (F3165)	WB (1:3000)	http://www.sigmaaldrich.com/catalog/product/sigma/f3165?lang=zh&region=CN
**DDB1**	Epitomics (3821-1)	WB (1:10000)	http://www.epitomics.com/products/product_info/2283

Supplementary Table S3. Quality control of RNA-seq results.

Rename	Total reads	Mapping efficiency	Uniquely Mapping efficiency	ERCC rate
WT-Zygote-1	16,624,350	90.00%	79.56%	1.406%
WT- Zygote -2	16,365,776	91.10%	83.45%	1.564%
WT- Zygote -3	19,152,313	88.00%	79.28%	1.561%
WT-2Cell-1	18,667,509	89.12%	65.37%	4.342%
WT-2Cell-2	17,762,840	87.72%	64.26%	4.431%
WT-2Cell-3	17,141,186	87.50%	64.91%	4.430%
siTut4/7-2Cell-1	17,376,236	89.50%	75.63%	2.846%
siTut4/7-2Cell-2	16,205,656	87.80%	71.38%	2.599%
siTut4/7-2Cell-3	15,556,924	87.30%	73.90%	2.602%

Supplementary Table S4. The cited published datasets

Title	Authors	Journal (year)	Paper website	License and accessibility
Allelic reprogramming of the histone modification H3K4me3 in early mammalian development	Bingjie Zhang, et al	Nature (2016) (1)	https://www.nature.com/articles/nature19361	GSE71434
Oocyte-expressed yes-associated protein is a key activator of the early zygotic genome in mouse	Chao Yu, et al	Cell Research (2016) (2)	https://www.nature.com/articles/cr201620	GSE74344
CNOT6L couples the selective degradation of maternal transcripts to meiotic cell cycle progression in mouse oocyte	Qianqian Sha, et al	EMBO Journal (2018) (3)	http://emboj.embopress.org/content/37/24/e99333.long	GSE118564

References

1. Zhang, B., Zheng, H., Huang, B., Li, W., Xiang, Y., Peng, X., Ming, J., Wu, X., Zhang, Y., Xu, Q. *et al.* (2016) Allelic reprogramming of the histone modification H3K4me3 in early mammalian development. *Nature*, **537**, 553-557.
2. Yu, C., Ji, S.Y., Dang, Y.J., Sha, Q.Q., Yuan, Y.F., Zhou, J.J., Yan, L.Y., Qiao, J., Tang, F. and Fan, H.Y. (2016) Oocyte-expressed yes-associated protein is a key activator of the early zygotic genome in mouse. *Cell Res*, **26**, 275-287.
3. Sha, Q.Q., Yu, J.L., Guo, J.X., Dai, X.X., Jiang, J.C., Zhang, Y.L., Yu, C., Ji, S.Y., Jiang, Y., Zhang, S.Y. *et al.* (2018) CNOT6L couples the selective degradation of maternal transcripts to meiotic cell cycle progression in mouse oocyte. *EMBO J*.
